# In mouse and *in vitro* models, bowel preparation promotes pathogen colonization, translocation, and exacerbation of inflammation

**DOI:** 10.1016/j.xcrm.2025.102517

**Published:** 2025-12-22

**Authors:** Charlotte A. Clayton, Imogen Porter, Brian D. Deng, Giselle McCallum, Apsara Srinivas, Claire Sie, Jerry Y. He, Alexander D. Pei, Dominique Tertigas, Deanna M. Pepin, Touran Fardeen, Katharine M. Ng, Sidhartha R. Sinha, Michael G. Surette, Bruce A. Vallance, Carolina Tropini

**Affiliations:** 1 Department of Microbiology & Immunology, University of British Columbia, Vancouver, BC V6T1Z3, Canada; 2 Genome Science & Technology, University of British Columbia, Vancouver, BC V6T1Z4, Canada; 3 Biochemistry and Biomedical Sciences, McMaster University, Hamilton, ON L8S4L8, Canada; 4 Department of Medicine, Division of Gastroenterology and Hepatology, Stanford University School of Medicine, Stanford, CA 94305, USA; 5 Department of Medicine, McMaster University, Hamilton, ON L8N3Z5, Canada; 6 Department of Pediatrics, Division of Gastroenterology, Hepatology and Nutrition, British Columbia Children’s Hospital Research Institute, Vancouver, BC V6H3V4, Canada; 7 School of Biomedical Engineering, University of British Columbia, Vancouver, BC V6T2B9, Canada; 8 Humans and the Microbiome Program, Canadian Institute for Advanced Research (CIFAR), Toronto, ON M5G1M1, Canada

**Keywords:** bowel preparation, gut microbiota, pathogen colonization, osmolality, *Salmonella enterica* serovar Typhimurium, polyethylene glycol, PEG, inflammatory bowel disease, IBD, pathobionts, translocation, humanized mice, gut-on-a-chip, DSS, IBD mouse model

## Abstract

In the United States, an estimated 14 million colonoscopies are performed yearly, each requiring patients to undergo bowel preparation, a laxative cleansing of the intestine’s luminal contents. Despite its widespread use, the effects of bowel preparation on gut physiology and susceptibility to pathogens remain poorly understood, particularly in individuals with compromised gut health. Using mouse and *in vitro* models, we find that bowel preparation with the laxative polyethylene glycol rapidly disrupts the gut, transiently increasing susceptibility to infection by *Salmonella* Typhimurium, including a non-motile mutant, and by gut pathobionts derived from ulcerative colitis microbiota. Bowel preparation also facilitates bacterial translocation to extraintestinal sites (mesenteric lymph nodes, liver, and spleen) and exacerbates inflammation in a chemically induced colitis model. Although these findings are preclinical, they suggest that bowel preparation may have underappreciated risks in vulnerable populations and warrant further clinical investigation.

## Highlights

• Bowel preparation with PEG transiently disrupts the mouse gut environment
• Susceptibility to *Salmonella* Typhimurium infection is increased post bowel prep
• Chemically induced colitis symptoms are exacerbated post bowel prep

## Graphical abstract

**Figure:**
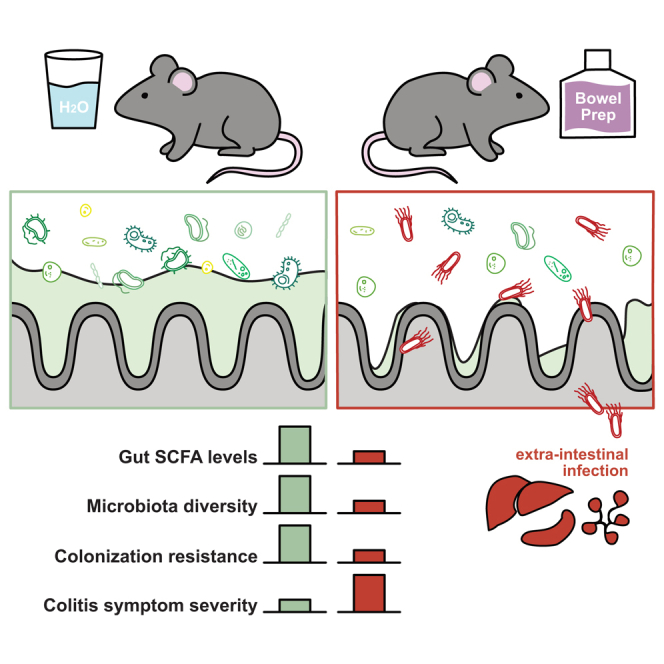

## Teaser

Bowel preparation is a widely used intensive laxative cleansing of the gut prior to colonoscopy. Clayton, Porter et al. demonstrate that this process rapidly and transiently disrupts the gut environment, increasing susceptibility to pathogen infection and exacerbating colitis in an IBD model.

## Introduction

In the United States, an estimated 14 million colonoscopies are performed yearly,^1^ each requiring patients to undergo bowel preparation (prep), a laxative cleansing of the intestine’s luminal contents. Bowel prep has been shown to cause short-term disruptions to the gut microbiota within the first several days post-procedure.^2^^,^^3^ While bowel prep is widely used and generally considered safe for routine screening colonoscopies, its transient effects on the gut environment, especially in individuals with altered microbiota, remain incompletely understood. Recent studies have shown that colonoscopies have significantly higher rates of infection compared to other screening procedures, with some centers reporting rates of 7-day post-endoscopic infections as high as 132 per 1,000 procedures^4^ and a 9.38-fold increased risk of infection compared to controls that did not undergo these procedures.^5^ However, these studies rely on retrospective insurance databases and cannot distinguish whether infections arise from the procedure itself, from interventions such as biopsy, or from preparatory steps like bowel prep. These studies, while limited by retrospective design and confounding variables, suggest a need for further mechanistic investigation into potential transient vulnerabilities. Here, we investigate whether bowel prep alone is sufficient to induce changes in the intestinal environment that could transiently reduce colonization resistance, using reductionist mouse and *in vitro* models to disentangle its specific effects.

Understanding the effects of bowel prep is especially important in vulnerable populations, including patients with inflammatory bowel disease (IBD), comprising both Crohn’s disease (CD) and ulcerative colitis (UC). The gut microbiota of patients with IBD often harbor microbial species known as pathobionts that can act as pathogens under certain conditions and are thought to worsen inflammation in IBD.^6^^,^^7^ Bowel prep may contribute to such disruptions, as reports have described IBD exacerbation and increased risk of sepsis and infection following colonoscopy.^4^^,^^8^ Consistent with these findings, our recent preprint analyzing a national database of quiescent IBD patients undergoing surveillance colonoscopy found an increased likelihood of post-colonoscopy steroid prescriptions, suggesting a risk of delayed symptom exacerbation.^9^ Previous studies have shown bowel prep may differentially affect the gut microbiota in patients with or without IBD, leading to long-term changes in microbiota composition several weeks after the procedure,^8^^,^^10^ as well as adverse effects such as toxic megacolon and increased emergency room visits that have not been mechanistically linked to the procedure itself.^11^^,^^12^^,^^13^^,^^14^^,^^15^^,^^16^ Understanding how bowel prep affects pathobiont growth and inflammatory state in this population is critical, as patients with IBD undergo more frequent colonoscopies than the general population to monitor disease progression.^17^

In previous work in mice, we showed that long-term (multi-day), low-concentration exposure to the laxative polyethylene glycol (PEG), used as an over-the-counter laxative and in human bowel prep, compromises the integrity of the intestinal mucus layer,^18^ an essential structure for preventing bacterial invasion of the mucosa. Moreover, PEG alters the gut microbiota and selects for bacterial species that can thrive in high-osmolality environments.^18^^,^^19^ Together, these changes have been associated with increased pathogen susceptibility in both mice and humans in some settings.^20^^,^^21^^,^^22^ Although previous studies have examined low-dose, long-term PEG exposure, the specific effects of clinically relevant short-term, high-dose bowel prep on pathogen susceptibility, particularly in vulnerable populations such as patients with IBD, remain poorly understood.

To address this gap, we asked two key questions: (1) How does bowel prep alter the gut microbiota and the intestinal environment? (2) Does bowel prep worsen host disease state in the context of pathobionts associated with IBD and inflammation? We hypothesize that bowel prep creates an environment in the gut that facilitates the growth and colonization of osmotically resistant pathogens and pathobionts, potentially increasing disease activity.

To answer these questions, we established mouse and *in vitro* models of bowel prep to investigate the effects of acute, high-dose laxative treatment with a time and spatial resolution not achievable in human studies. We examined the impacts of bowel prep on the gut microbiota, the local intestinal environment, and host resistance against pathogen colonization by *Salmonella enterica* serovar Typhimurium (*Salmonella* Typhimurium). We found that bowel prep with PEG transiently altered the gut environment in multiple ways, temporarily increasing its susceptibility to colonization by *Salmonella* Typhimurium, including a non-motile mutant normally unable to invade the gut. In addition, gut preparation promoted the translocation of these bacteria to lymph nodes, liver, and spleen. In a human IBD microbiota colitis model, bowel prep worsened colitis severity, suggesting that bowel prep can transiently lower host defenses in the context of intestinal inflammation. These results highlight bowel prep as a tractable experimental model for dissecting colonization resistance and suggest that, under certain conditions, it may transiently reduce host defenses in ways that warrant further investigation, particularly in individuals with microbiota rich in pathobionts.

## Results

### Bowel prep disrupts intestinal osmolality, the mucus layer, and short-chain fatty acid levels in the mouse gut

**Figure 1:**
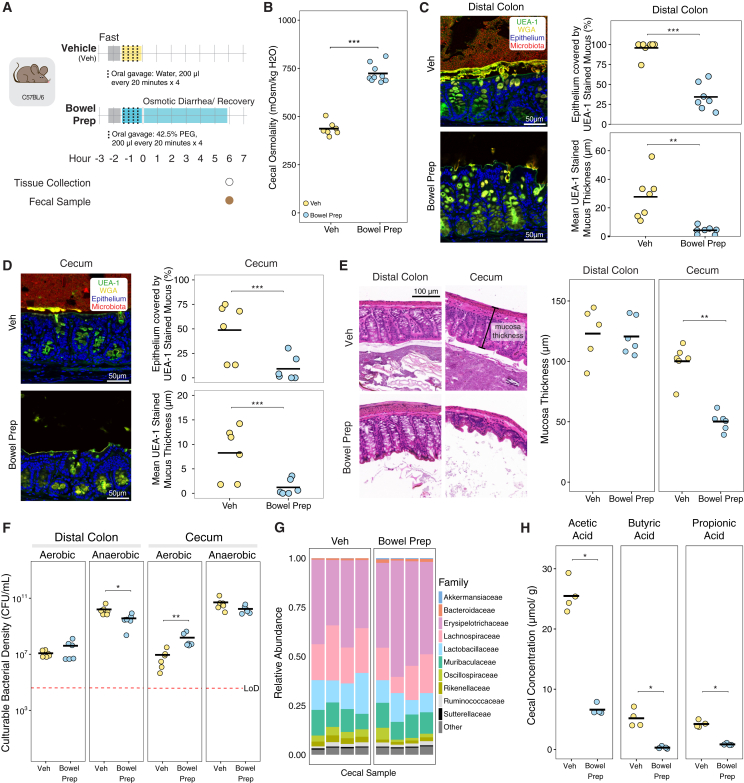
Bowel preparation with a laxative, polyethylene glycol, disrupts intestinal osmolality, the mucus layer, and short-chain fatty acid levels 6 h post-procedure in the mouse gut (A) Schematic representation of our mouse model for bowel preparation (prep). Mice were orally gavaged four times with 42.5% PEG in water (bowel prep) or water (vehicle [Veh]) at 20-min intervals. (B) Cecal osmolality in bowel prep- vs. vehicle-treated mice (Veh *n* = 7, bowel prep *n* = 9). (C and D) Left: 6 h post-treatment, a representative confocal micrograph of the distal colon and cecum, respectively, shows the mucus layer (staining with UEA-1 [green] and WGA [yellow]). Epithelial cell nuclei (blue) were stained with DAPI. Right: percentage of epithelium covered by mucus (top) and mucus layer thickness (bottom), quantified using UEA-1 fluorescence (Veh *n* = 7, bowel prep *n* = 6, two independent experiments). (E) Left: representative images of hematoxylin and eosin (H&E)-stained sections of the distal colon and cecum 6 h post-treatment. Right: colon and cecum mucosa thickness in the stained sections (Veh *n* = 6, bowel prep *n* = 6). (F) Loads of culturable anaerobic bacteria 6 h post-treatment in the distal colon and cecum (Veh *n* = 6, bowel prep *n* = 6). (G) 16S rRNA sequencing of the contents of the cecum 6 h post-treatment. (H) Abundance of three SCFAs in the cecum 6 h post-treatment, measured using gas chromatography-mass spectrometry (Veh *n* = 4, bowel prep *n* = 4). Statistics: for all comparisons, statistical significance was assessed using the Wilcoxon rank-sum test. *p* > 0.05; ns (not significant, not shown), *p* < 0.05∗, *p* < 0.01∗∗, *p* < 0.001∗∗∗, *p* < 0.0001∗∗∗∗. All points show biological replicates. Abbreviation: LoD, limit of detection.

We hypothesized that bowel prep would cause a significant disruption of the gut environment, leading to reduced bacterial abundance and depletion of key microbial metabolites. To mimic the human bowel prep process, we orally gavaged C57BL/6J mice with PEG (bowel prep) or water (vehicle) four times at 20-min intervals (Figure 1A) and assessed changes to the cecal and colon environments 6 h later. Bowel prep-treated mice had an average cecal osmolality that was 1.7-fold higher than vehicle-treated controls (724 vs. 438 mOsm/kg, *p* = 0.00017; Figure 1B), a higher cecal mass indicative of osmotic diarrhea, and increased water excretion during bowel prep (Figure S1A). In fixed, stained, and imaged tissue sections, the distal colon of vehicle-treated mice displayed the expected thick, continuous mucus layer (Figure 1C). Conversely, bowel prep-treated mice revealed a largely depleted mucus layer (Figure 1C) and loss of luminal contents including bacteria (Figure S1B) in the colon, despite having similar mass (Figure S1A). Both the percentage of distal colon epithelium covered by mucus and the average mucus thickness were significantly reduced (Figures 1C and S1C). Upstream in the cecum, the mucus layer in vehicle-treated mice was more hydrated and patchier than in the distal colon, as expected^23^^,^^24^; the percentage coverage and average thickness were also significantly lower (Figure 1D). These findings indicate that even short-term, high-concentration PEG exposure can severely disrupt the protective mucus barrier in the gut.

We wondered whether the disruption of the protective mucus layer might lead to abnormalities in the tissue. We assessed pathology in hematoxylin and eosin (H&E)-stained sections of the cecum and distal colon, including in the lumen, epithelium, mucosa, and submucosa. No immune cell infiltration or difference in tissue pathology was observed in samples from bowel prep- or vehicle-treated mice (Figure S1D). However, in the cecum, mucosa thickness was significantly lower in bowel prep- vs. vehicle-treated mice (Figure 1E). This reduction in mucosa thickness was not observed in the distal colon (Figure 1E). This suggests that bowel prep-induced excretion of water, which reduces mucosa thickness, is not uniform throughout the gut.

Next, we measured how bowel prep treatment affects the gut microbiota in mice. In the distal colon and small intestine, culturable anaerobic bacterial loads were lower in bowel prep- vs. vehicle-treated mice; however, no difference was observed in the cecum (Figures 1F and S1E). By contrast, culturable aerobic bacterial loads in the distal colon and small intestine—although representing a much smaller fraction of the total counts—did not differ between the two treatment groups. In the cecum, they were 17-fold higher in bowel prep- vs. vehicle-treated mice (Figures 1F and S1E). These findings indicate an overall reduction in microbial load due to bowel prep, and the expansion of aerobic bacteria, consistent with oxygenation of the gut environment.^25^ The composition of the cecal microbiota 6 h post-treatment, as measured by 16S rRNA sequencing, was largely similar in bowel prep- vs. vehicle-treated mice (Figures 1G and S1H). Combined with the bacteria load data, this observation suggests that 16S rRNA sequencing is measuring a portion of the microbiota that is either not culturable or no longer alive post-bowel prep.

To test whether changes in microbial load led to lower levels of microbial metabolites, we measured individual short-chain fatty acid (SCFA) levels in the cecum’s contents, a major site of microbial activity in the mouse intestine. SCFA levels were significantly lower in bowel prep- vs. vehicle-treated mice (Figures 1H and S1F). We also measured the pH of the cecal contents as it is affected by SCFA levels and can impact bacterial growth and composition^19^; however, cecal pH did not significantly differ between the treatment groups (Figure S1G). This suggests that while bowel prep reduces SCFA production, residual levels or buffering by other compounds may be sufficient to maintain luminal pH within a relatively stable range.

We next tested whether bowel prep altered intestinal permeability. FITC-dextran gavage and serum analysis showed no significant difference between bowel prep and control mice (Figure S1I).

Altogether, these findings support the hypothesis that bowel prep leads to a significant disruption of the gut environment and impairs microbial metabolism.

### Bowel prep promotes *Salmonella* Typhimurium colonization, translocation, and pathology in mice

**Figure 2:**
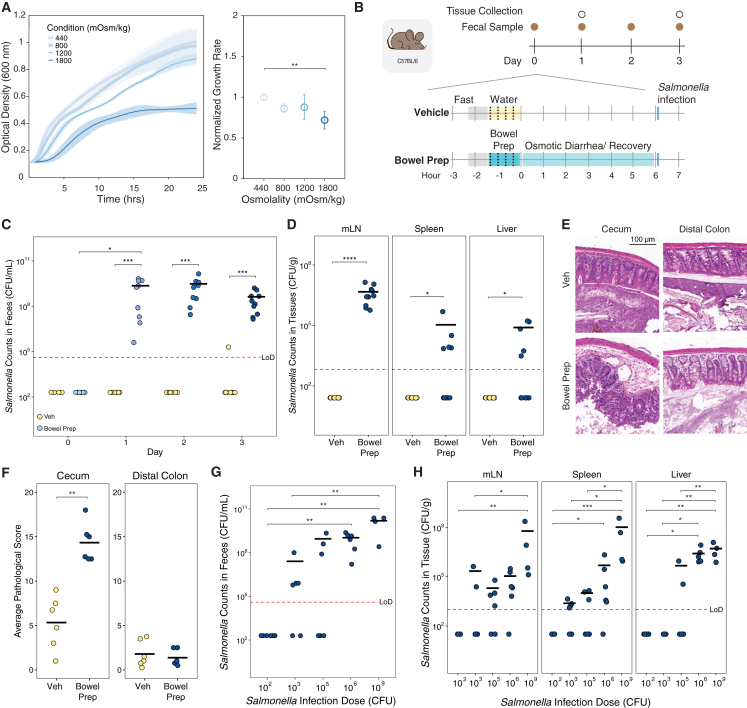
Bowel prep promotes *Salmonella enterica* serovar Typhimurium colonization, translocation, and gut pathology in mice (A) *Salmonella* Typhimurium growth rates under normal (∼450 mOsm/kg) and osmotic conditions comparable to those in the post-bowel prep gut of mice (∼800 mOsm/kg; each osmolality *n* = 4). (B) Schematic of the bowel prep-pathogen mouse model. Mice were infected with *Salmonella* Typhimurium 6 h after bowel prep with PEG. (C) *Salmonella* Typhimurium counts in the feces of bowel prep- vs. vehicle-treated mice (Veh *n* = 8, bowel prep *n* = 10). (D) *Salmonella* Typhimurium translocation levels from the gut to the mesenteric lymph nodes (mLN), liver, and spleen 3 days after inoculation (Veh *n* = 8, bowel prep *n* = 10). Counts in the extraintestinal organs were measured via plating. (E) Representative images of H&E-stained sections of the distal colon and cecum in bowel prep- vs. vehicle-treated mice inoculated with 10^6^ CFU of *Salmonella* Typhimurium. (F) Histopathological scores of the distal colon and cecum sections from (E) (Veh *n* = 6, bowel prep *n* = 6). (G) Fecal levels of *Salmonella* Typhimurium inoculated with different doses (100–10^6^ CFU *n* = 6, 10^9^ CFU *n* = 4) 3 days post-treatment with bowel prep. (H) *Salmonella* Typhimurium translocation levels from the gut to the mLN, spleen, and liver in bowel prep-treated mice 3 days post-bowel prep in the mice from (G). **Statistics**: differences between multiple groups were analyzed by one-way ANOVA with Tukey’s post hoc test (A). Differences between two groups were analyzed by Wilcoxon rank-sum test (C–H). Differences within groups at different time points were analyzed by Friedman test followed by Nemenyi post hoc test (C). *p* > 0.05; ns (not significant, not shown), *p* < 0.05∗, *p* < 0.01∗∗, *p* < 0.001∗∗∗, *p* < 0.0001∗∗∗∗. Error bars show standard deviation. Abbreviation: LoD, limit of detection.

Having observed that bowel prep treatment reduces the thickness and coverage of the gut mucus layer and SCFA levels and increases intestinal osmolality (Figure 1B), we hypothesized that enteric pathogens would be able to bypass colonization resistance more easily in bowel prep- vs. vehicle-treated mice.^26^^,^^27^ Specifically, given that commensal microbiota members are highly sensitive to osmolality,^18^^,^^19^ we reasoned that an osmotically resilient pathogen may be advantaged during bowel prep. We measured growth rates of *Salmonella* Typhimurium, an enteric pathogen known to withstand osmotic stress in high-salt environments,^28^ in growth media adjusted with PEG to various osmolality levels. At an osmolality comparable to the post-bowel prep gut of mice (∼800 mOsm/kg), *Salmonella* Typhimurium growth was indistinguishable from normal osmotic conditions (∼450 mOsm/kg; Figure 2A). Next, we challenged bowel prep- and vehicle-treated mice with a standard infectious dose of 10^6^ CFU *Salmonella* Typhimurium via oral gavage (Figure 2B). Whereas vehicle-treated mice showed no detectable levels of *Salmonella* Typhimurium in their feces post-treatment, bowel prep-treated mice showed high levels of colonization, which persisted at least 3 days post-inoculation, indicating colonization and expansion in the intestinal tract (Figure 2C). This finding was not sex dependent (Figure S2A). These results support our hypothesis that bowel prep-induced disruption can facilitate pathogen colonization and growth.

Next, we tested whether *Salmonella* Typhimurium could translocate from the gut. 3 days post-inoculation, bowel prep-treated mice showed significant colonization of the mesenteric lymph nodes (mLN), spleen, and liver (Figure 2D), whereas no colonization occurred in vehicle-treated controls, indicating that translocation is rare under normal conditions.

We also used H&E staining to visualize the host tissue responses to *Salmonella* Typhimurium 3 days post-infection. In the cecum, combined histopathological scores for the lumen, epithelium, mucosa, and submucosa were significantly higher in bowel prep- vs. vehicle-treated mice (Figures 2E and 2F). No significant difference in histopathological scores was found for the distal colon, suggesting that during bowel prep the cecum was the primary site of infection.

We next compared the efficacy of *Salmonella* Typhimurium colonization in bowel prep to the field-standard model, which involves pre-treating mice with antibiotics to deplete colonization resistance by native gut microbiota.^29^ 1 day after infection, the maximum fecal burden of *Salmonella* Typhimurium was 11-fold higher in streptomycin- vs. bowel prep-treated mice (Figure S2B). This burden persisted for 3 days after infection (Figure S2C). However, *Salmonella* Typhimurium levels in the extraintestinal organs were similar between the streptomycin- and bowel prep-treated mice (Figures 2D and S2D), and pathology was observed in both models at comparable levels (Figures 2E, 2F, S2E, and S2F). Altogether, these findings indicate that bowel prep alone, without antibiotics, can facilitate expansion of *Salmonella* Typhimurium in the gut and its translocation to extraintestinal sites.

Finally, we investigated the minimum dose required for *Salmonella* Typhimurium to colonize the gut of mice subjected to bowel prep. We hypothesized bowel prep treatment would create a gut environment vulnerable to even low levels of pathogen. Supporting this hypothesis, among bowel prep-treated mice, inoculation with as few as 1,000 CFU led to most mice becoming colonized (Figure 2G). In addition, in bowel prep-treated mice, *Salmonella* Typhimurium translocation levels 3 days after inoculation largely increased with the size of the dose administered (Figure 2H). These findings indicate that a much lower minimum dose of *Salmonella* Typhimurium is required to colonize the gut after bowel prep, and the higher the dose, the higher the level of translocation to extraintestinal organs.

### Gut resistance to *Salmonella* Typhimurium recovers over time after bowel prep

**Figure 3:**
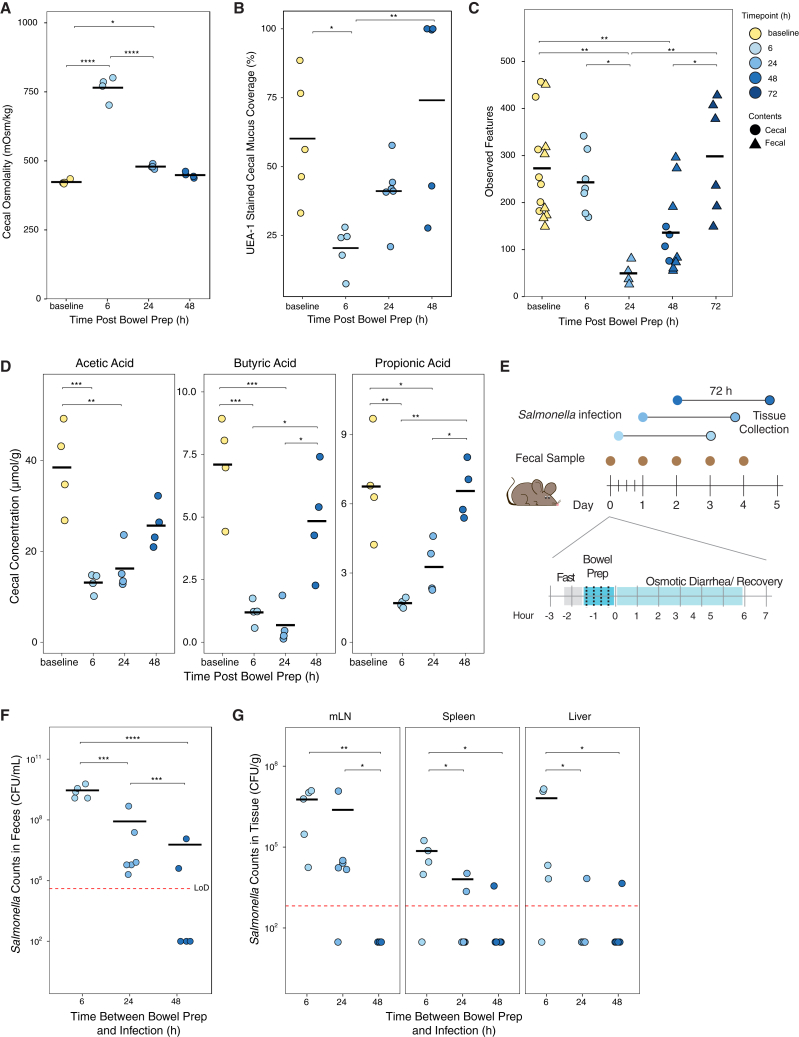
Gut susceptibility to *Salmonella* Typhimurium is reduced by 24 h after bowel prep (A) Cecal osmolality at different time points after bowel prep with PEG (each time point *n* = 4). (B) Percent mucus coverage of the cecal epithelium, as measured with UEA-1 staining (baseline, 6 h, 48 h *n* = 5, 24 h *n* = 6, three independent experiments). (C) Number of observed features (alpha diversity) in the cecal and fecal microbiome, determined from 16S rRNA sequencing (baseline *n* = 14, 6 h *n* = 7, 24 h *n* = 4, 48 h *n* = 11, 72 h *n* = 6, four independent experiments). (D) Abundance of three SCFAs in the cecum, measured by gas chromatography-mass spectrometry (each time point *n* = 4). (E) Schematic of bowel prep-pathogen mouse model with *Salmonella* Typhimurium inoculation at 6, 24, and 48 h after bowel prep treatment. (F) Fecal levels of *Salmonella* Typhimurium 72 h post-inoculation (6 h *n* = 5, 24 h *n* = 6, 48 h *n* = 5, two independent experiments). (G) *Salmonella* Typhimurium translocation from the gut to the mLN, liver, and spleen (6 h *n* = 5, 24 h *n* = 6, 48 h *n* = 5, two independent experiments). Culturing of organs to obtain pathogen counts was performed 72 h post-inoculation. Statistics: comparisons between groups were performed by Student’s *t* test (A), one-way ANOVA with Tukey’s post hoc test (C), or Kruskal-Wallis followed by Dunn’s post hoc test (D–G). *p* > 0.05; ns (not significant, not shown), *p* < 0.05∗, *p* < 0.01∗∗, *p* < 0.001∗∗∗, *p* < 0.0001∗∗∗∗. Abbreviation: LoD, limit of detection.

We next investigated which physiological differences might be responsible. Having observed that bowel prep treatment significantly increased pathological scores in the cecum (Figures 2E and 2F), we characterized the cecal environment in mice at different timepoints post-bowel prep. Cecal osmolality after bowel prep treatment (765 mOsm/kg) recovered rapidly; 24 h after bowel prep, values were similar to those at baseline (479 vs. 423 mOsm/kg, *p* = 0.02; Figure 3A). Cecal mucus coverage and thickness also recovered to baseline values 48 h after bowel prep (Figures 3B and S3A–S3C). Furthermore, the cecal and fecal microbiota were depleted by bowel prep, with species diversity levels recovering by 72 h (Figures 3C and S3E). Microbiota composition was similarly disrupted (Figure S3F). Finally, SCFA concentrations, particularly for butyrate, remained depleted in bowel prep-treated mice 24 h after treatment but recovered by 48 h (Figures 3D and S3D).

To investigate whether these changes impacted resistance to pathogen colonization, we challenged mice with 10^6^ CFU *Salmonella* Typhimurium 6, 24, and 48 h after bowel prep treatment (Figure 3E). 72 hours after inoculation, fecal *Salmonella* Typhimurium levels were highest in mice inoculated 6 h after bowel prep and progressively lower when inoculated at 24 or 48 h (Figure 3F). Levels of extra-intestinal *Salmonella* Typhimurium were similarly highest in mice inoculated 6 h post-bowel prep, reaching undetectable levels in the 48-h inoculation group (Figure 3G). These data suggest that bowel prep recovery begins within 48 h and that microbiota-related factors play an important role in resistance against colonization.

### Flagellar motility is not required for *Salmonella* Typhimurium to colonize the gut or translocate to mesenteric lymph nodes after bowel prep in mice

**Figure 4:**
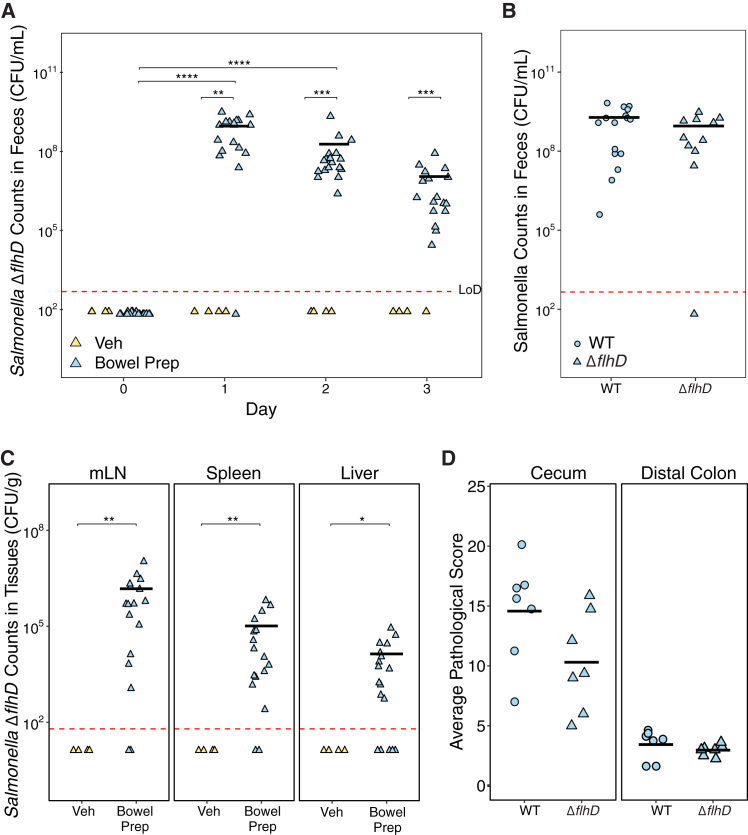
Flagellar motility is not required for *Salmonella* Typhimurium mutant *ΔflhD* gut colonization, translocation to the mesenteric lymph nodes, or pathology in mice subjected to bowel prep (A) *Salmonella* Typhimurium *ΔflhD* colonization levels in mice inoculated 6 h post bowel prep- or vehicle treatment (Veh *n* = 4, bowel prep *n* = 17, two independent experiments for bowel Prep and one for Veh). (B) Wild-type (WT) and *Salmonella* Typhimurium *ΔflhD* counts in the feces 1 day after inoculation (WT *n* = 16, *ΔflhD n* = 17, two independent experiments). (C) *Salmonella* Typhimurium *ΔflhD* levels in mesenteric lymph nodes (mLN), spleen, and liver 72 h after bowel prep (Veh *n* = 4, bowel prep *n* = 17, two independent experiments for bowel prep and one for Veh). (D) Histopathological scores (maximum possible score of 24) of the distal colon and cecum sections from bowel-prepped mice (WT *n* = 7, *ΔflhD n* = 7). Statistics: Comparisons between treatment groups were measured using the Wilcoxon rank-sum test and within groups at different time points with a Friedman test followed by Nemenyi post hoc test. *p* > 0.05; ns (not significant, not shown), *p* < 0.05∗, *p* < 0.01∗∗, *p* < 0.001∗∗∗, *p* < 0.0001∗∗∗∗. Abbreviation: LoD, limit of detection.

Normally, flagellar propulsion is critical for *Salmonella* Typhimurium and other bacterial pathogens to penetrate the mucus layer of the gut and infect the underlying epithelium.^30^^,^^31^ However, given the defects in the cecal mucus layer that we observed after bowel prep (Figure 3B), we hypothesized that a non-motile *Salmonella* Typhimurium mutant might be able to colonize post-bowel prep. We challenged mice with a non-motile *Salmonella* Typhimurium *ΔflhD* mutant 6 h after bowel prep or vehicle treatment. The *ΔflhD* mutant is missing the gene responsible for control of flagellar production^32^ but, similar to wild-type *Salmonella* Typhimurium, is resilient to osmotic perturbation (Figure S4). Consistent with our hypothesis, *Salmonella* Typhimurium *ΔflhD* efficiently colonized the gut only in bowel prep-treated mice, and its fecal levels were comparable to those of wild-type *Salmonella* Typhimurium 1 day after inoculation (Figures 4A and 4B). The *ΔflhD* mutant was also able to translocate from the gut to the mLN, liver, and spleen in bowel prep-treated mice but not vehicle-treated mice (Figure 4C). These observations indicate that bowel prep-induced disruption allows *Salmonella* Typhimurium to colonize the gut and translocate to extra-intestinal organs without the normal requirement for motility. Consistent with this, H&E histopathology scores did not significantly differ between mice infected with the Δ*flhD* mutant vs. the wild type (Figure 4D), although a non-significant trend was observed. This supports the idea that the loss of the mucus barrier after bowel prep may reduce the necessity of flagellar motility.

### Bowel prep induces a small but coordinated cecal tissue transcriptional response

**Figure 5:**
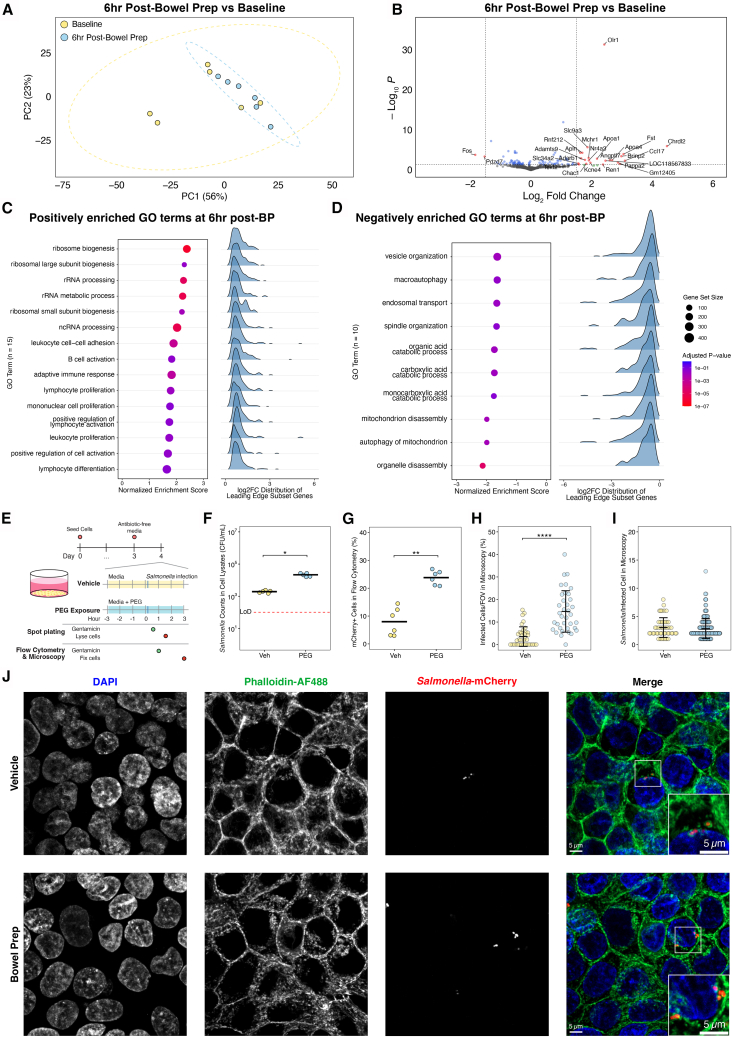
Bowel prep induces mild changes in epithelial gene expression *in vivo*, while PEG promotes *Salmonella* Typhimurium invasion *in vitro* (A) Principal component analysis plot of mouse cecal gene expression at baseline vs. 6 h post-bowel prep treatment, assessed by PERMANOVA and PERMDISP. (B) Volcano plot of differential expression in mouse cecal tip tissue at baseline vs. 6 h post-bowel prep compared to baseline. Labeled genes have adjusted *p* values (Benjamini-Hochberg false discovery rate-corrected) < 0.05 and log_2_ fold change > 1.5. (C and D) Gene set enrichment analysis of Gene Ontology (GO) Biological Process terms on differentially expressed genes at baseline vs. 6 h post-bowel prep vs. baseline. Dot plots display normalized enrichment scores (NESs), gene set size and color indicate adjusted *p* values (Benjamini-Hochberg false discovery rate-corrected), while ridge plots show the log_2_ fold-change distributions of leading-edge subset genes. (C) Significantly positively enriched GO terms (NES > 0). (D) Significantly negatively enriched GO terms (NES < 0). (E) Schematic of the *in vitro* PEG exposure model. (F) Intracellular *Salmonella* Typhimurium counts in cell lysates (*n* = 5 for each condition). (G) Proportion of cells with mCherry-positive signal from flow cytometry (*n* = 6 per condition from two independent experiments). (H) Infected cells per field of view (FOV) from microscopy images (*n* = 32 FOVs per condition from three independent experiments). (I) *Salmonella* Typhimurium counts in infected cells (Veh *n* = 46, bowel prep *n* = 193 from the cells in [H]). (J) Representative confocal micrographs of fluorescently labeled HT-29 cells infected with mCherry-*Salmonella* Typhimurium (red) and stained with DAPI (blue) to visualize nuclei and phalloidin-AF488 *(*green) to visualize filamentous actin. Statistics: comparisons between treatment groups were analyzed using a Wilcoxon rank-sum test (F and G) or *t* test (H and I). Black bars show the mean and standard deviation. *p* > 0.05; ns (not significant, not shown), *p* < 0.05∗, *p* < 0.01∗∗, *p* < 0.001∗∗∗, *p* < 0.0001∗∗∗∗.

We next asked whether host tissue responses might contribute to increased pathogen susceptibility. We performed bulk RNA sequencing and analyzed the transcriptome of mouse cecal tissue at baseline and 6 h post-bowel prep. Groups were not significantly separated in principle component analysis clustering of the cecal gene expression of bowel prep-treated mice (R^2^ = 0.14, *p* = 0.098) or dispersed (*p* = 0.492) based on treatment (Figure 5A). Only 25 genes were significantly differentially expressed (Figure 5B). Of the top 50 differentially expressed genes by absolute *Z* score, few were related to mucus production and barrier function (Figure S5A), and individual genes related to mucus production, barrier function, and immune system processes showed no significant changes between baseline and bowel prep (Figure S5B).

We then performed pathway-level analysis. We discovered that at 6 h post-bowel prep 15 Gene Ontology (GO) Biological Process terms were significantly positively enriched (normalized enrichment score [NES] > 0), including terms related to protein synthesis and adaptive immune system functions (Figure 5C; Table S3). We found 10 significantly negatively enriched GO terms (NES < 0), including organelle recycling processes, intracellular trafficking processes, and catabolic functions (Figure 5D; Table S3). Positively enriched terms shared overlapping core enrichment genes, largely among pathways related to protein synthesis and immune activation (Figure S5C), while negatively enriched terms overlapped in genes associated with organelle degradation and catabolic processes (Figure S5D). Although individual fold changes were modest, the significant NES indicates a coordinated shift in gene expression across these pathways.

Together, these analyses suggest that bowel prep triggers an early but modestly scaled, coordinated transcriptional response in cecal tissue, characterized by upregulation of protein synthesis and immune pathways alongside downregulation of catabolic processes.

### PEG exposure promotes *Salmonella* Typhimurium invasion in a human intestinal epithelial cell model

Because the transcriptional response in cecal tissue suggested only modest host changes, we asked whether bowel prep conditions directly alter epithelial susceptibility to bacterial invasion using *in vitro* human intestinal epithelial cell models. We treated confluent monolayers of HT-29 cells, an established human intestinal epithelium model,^33^^,^^34^ with PEG-supplemented media for 3, 6, or 24 h (Figure S6A). To model *in vivo* osmolality changes between vehicle- and bowel prep-treated mice (Figure 1B), we adjusted cell culture media from 350 mOsm/kg to 600 mOsm/kg, with additional testing at 900 and 1200 mOsm/kg. We infected monolayers in these conditions with *Salmonella* Typhimurium for 90 min and quantified intracellular bacteria from cell lysates. Invasion was maximal at the 600 mOsm/kg condition, with intracellular counts increasing 6.9-fold from 1 h (4 × 10^4^ CFU/mL) to 3 h (2.75 × 10^5^ CFU/mL) of pre-treatment (Figure S6B). Compared to the vehicle, monolayers pretreated for 3 h at 600 mOsm/kg showed an 11.6-fold increase in mean bacteria counts (Figure 5F). These results indicate that short-term exposure to PEG substantially increases epithelial susceptibility to bacterial entry.

To characterize the degree of intracellular invasion at the single-cell level, we pretreated HT-29 monolayers for 3 h at 600 mOsm/kg then infected cells with a constitutive mCherry-expressing *Salmonella* Typhimurium strain^35^ for 3 h to allow bacteria expansion (Figure 5E). Flow cytometry showed a 3-fold increase in the proportion of infected cells relative to vehicle controls (Figures 5G, S6C, and S6D), corroborated by confocal imaging (Figures 5H and S6E). Infected cells contained similar bacterial loads across conditions (Figure 5I), suggesting that bowel prep conditions may enhance bacterial entry but not intracellular replication. Phalloidin staining revealed modest actin disorganization after PEG treatment, a phenotype that warrants further investigation (Figure 5J).

To complement our monolayer invasion assays, we also tested the effects of bowel prep conditions in a gut-on-a-chip model that more closely recapitulates intestinal physiology.^36^ In this system, bowel prep treatment modestly increased epithelial permeability and *Salmonella* Typhimurium translocation compared to controls, though not significantly (Figures S6F–S6I). Together, our results suggest that PEG exposure enhances epithelial vulnerability to *Salmonella* Typhimurium invasion and translocation *in vitro*, consistent with the increased pathogen colonization and translocation *in vivo*.

### Bowel prep exacerbates colitis and enhances pathobiont translocation in human IBD microbiota-associated mice

In previous work, we systematically profiled the tolerance of 92 representative human gut bacterial strains to a range of osmotic conditions.^19^ We found that many abundant commensals, particularly strict anaerobes, exhibited markedly reduced growth at osmolalities comparable to those induced by bowel prep; conversely, Enterobacteriaceae members were among the most osmotolerant taxa.^19^ Given that *Salmonella* Typhimurium is highly osmotolerant and can disseminate after bowel prep, we next asked whether IBD-associated pathobionts might similarly thrive under these conditions. We examined the ability of 130 strains isolated from patients with UC that belonged to genera previously identified as pathobionts^37^^,^^38^ (family Enterobacteriaceae; genera: *Proteus*, *Morganella*, *Kluyvera*, *Klebsiella*, *Escherichia*, *Enterobacter*, and *Citrobacter*) for their growth across a range of osmolalities simulating bowel prep. These potential pathobionts showed strong growth at osmotic levels experienced in the mouse gut post-bowel prep (∼765 mOsm/kg) in both anaerobic and aerobic conditions (Figures S7A and S7B). As expected, most strains were able to grow above 1100 mOsm/kg, well above the levels many commensal bacteria are able to grow in.^19^ These results indicate that pathobiont genera can thrive under the high osmotic conditions created by bowel prep.

Next, we asked whether the pathobionts’ relatively high osmotic resistance could lead to a shift in community composition under conditions of continued elevated osmotic stress. We cultured a fecal sample obtained from an individual with ulcerative colitis (hereafter referred to as hIBD1) under osmotic stress levels simulating normal (400 mOsm/kg) and bowel prep-treated (800 mOsm/kg) conditions. We found a shift in community composition under the higher osmolality conditions (Figure S7C), including increased abundance of the family Enterobacteriaceae. Specifically, the abundance of the genera *Morganella* and *Escherichia-Shigella* increased (Figure S7D), indicating that even in a complex community, these potential IBD-associated pathobionts had a competitive advantage. We next tested whether bowel prep could promote the expansion and systemic dissemination of these bacteria in a mouse model of ulcerative colitis.

**Figure 6:**
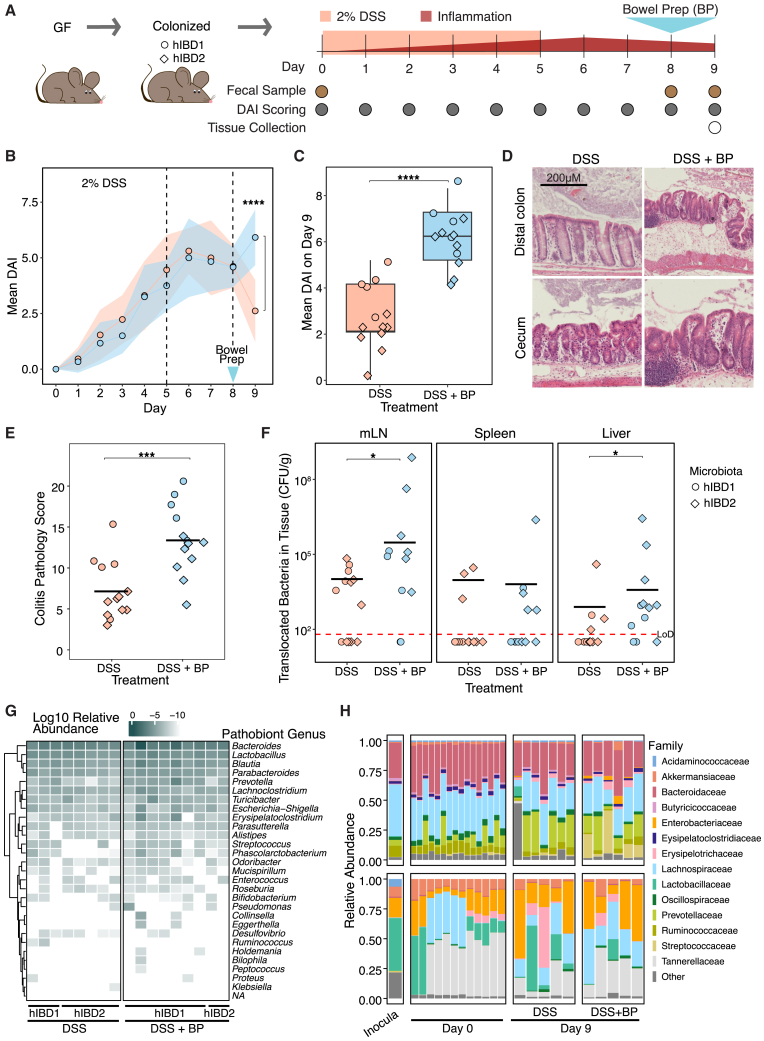
In human IBD microbiota-associated mice undergoing acute inflammation, bowel prep exacerbates colitis and increases extra-intestinal bacterial translocation (A) Schematic of the human microbiota colitis model. (B) Mean colitis disease activity index (DAI) of mice assessed daily, with bowel prep on day 8 (control *n =* 13, bowel prep *n =* 13). (C) DAI scores of individual mice on day 9. (D) Representative H&E-stained sections of the distal colon and cecum. (E) Colitis pathology scores in the distal colon. (F) Quantification of bacterial translocation from the gut to the mLN, liver, and spleen 1 day after hIBD mice received bowel prep. (G) 16S rRNA sequencing of bacteria translocated to the mLN of mice shows the presence of genera from pathobionts associated with IBD.^6^^,^^44^^,^^45^^,^^46^^,^^47^^,^^48^^,^^49^^,^^50^^,^^51^^,^^52^^,^^53^^,^^54^^,^^55^^,^^56^^,^^57^^,^^58^ (H) Stool 16S rRNA sequencing of mice colonized with hIBD1 (top) or hIBD2 (bottom) microbiotas before and after DSS treatment with and without bowel prep. Statistics: comparisons between groups analyzed using a Wilcoxon rank-sum test. *p* > 0.05; ns (not significant, not shown), *p* < 0.05∗, *p* < 0.01∗∗, *p* < 0.001∗∗∗, *p* < 0.0001∗∗∗∗. Abbreviation: LoD, limit of detection.

We first humanized germ-free mice with fecal samples from a healthy participant (hHealthy) or the characterized patient with ulcerative colitis (hIBD1) and found that there was no observable long-term translocation 2 weeks post-bowel prep (Figures S8A and S8B). This suggests that bowel prep by itself does not cause sustained bacterial translocation. Given reports of post-colonoscopy flare-ups in patients with IBD, we hypothesized that acute inflammation might affect the response to bowel prep in an IBD microbiota context. To test this, we colonized mice with two patient ulcerative colitis microbiota and a healthy participant microbiota (hIBD1, hIBD2, and hHealthy, respectively) and induced acute colitis by treating mice with 2% dextran sodium sulfate (DSS) in drinking water for 5 days (Figures 6A and S8C). We measured the disease activity index (DAI; a composite score of body weight loss, stool consistency, fecal bleeding, and behavior^39^^,^^40^^,^^41^) daily starting from the onset of DSS treatment. Both hIBD groups showed higher DAI than the healthy controls, with variability between donors, consistent with the microbiota dependence of DSS response^42^^,^^43^ (Figures S8D and S8E).

Because mice colonized with a healthy microbiota are not expected to develop spontaneous intestinal inflammation, bowel prep was performed only in hIBD-colonized mice. Bowel prep was performed 2 days after cessation of DSS (Figure 6A) to avoid treatment interference and to better mimic the clinical scenario in which colonoscopy is performed shortly after onset of inflammation. We found that hIBD mice treated with bowel prep showed a 2-fold increase in DAI 24 h post-treatment compared to the no-treatment controls (*p* = 0.00013, Figures 6B and 6C). This effect was preserved between donor groups, though the magnitude varied (Figure S8F). We then measured tissue pathology by performing H&E staining and scoring^59^ in the cecal tip and distal colon. Supporting the DAI results, we found that mice that received bowel prep had an average distal colon pathology score of 13.3 compared to untreated mice, which scored 7.1 (out of 24, *p* = 0.00041, Figures 6D and 6E).

Having measured significant increases in inflammation due to bowel prep, we then tested the translocation of bacterial species to extra-intestinal organs. We found that the amount of anaerobic extra-intestinal bacteria increased 30-fold in the mLN (*p* = 0.037) and 5-fold in the liver (*p* = 0.015) 24 h post-bowel prep compared to mice that received no treatment (Figure 6F). We then performed 16S rRNA sequencing of the mLN to identify which bacteria translocated. This analysis revealed the presence of many pathobiont species associated with IBD in the mLN independent of bowel prep (Figures 6G and S8H). While potential pathobiont species were found in both the bowel prep group and no-treatment controls likely due to the DSS treatment, the number of observed species trended higher in the bowel prep group (*p* = 0.11, Figure S8G). Finally, sequencing of stool samples collected at the start and end of the experiment showed little change in microbiota composition, which remained largely reflective of their starting inocula (Figure 6H).

Together, these experiments demonstrate that bowel prep exacerbates colitis in human IBD microbiota-associated mice and enables osmotolerant Enterobacteriaceae pathobionts to expand and translocate beyond the gut, highlighting a mechanism by which transient environmental disruption can precipitate both local and systemic disease.

## Discussion

In this study, we set out to use mouse and *in vitro* models of bowel prep to determine whether the procedure increases the gut’s vulnerability to colonization by pathogens. We showed that bowel prep with PEG (1) alters the intestinal environment (Figure 1), (2) eliminates the natural microbiota’s and epithelial protection against *Salmonella* Typhimurium invasion (Figures 2, 3, 4, and 5), and (3) exacerbates colitis in a human IBD microbiota-associated mouse model (Figure 6). Our findings show that bowel prep reduces the gut microbiota’s natural resistance to pathogens through multiple mechanisms, including increased levels of osmotic stress, depletion of the mucus layer, and reduced competition by commensal bacteria.

Shortly after bowel prep is performed, before the commensal microbiota and gut environment have a chance to recover, hosts are extremely vulnerable to pathogens. In this study, inoculation with as low as 1,000 *Salmonella* Typhimurium cells successfully colonized the gut of mice treated with bowel prep 6 h earlier and disseminated to the lymph nodes, spleen, and liver (Figures 2G and 2H). This dose is between ten thousand and a million times lower than typically used in the streptomycin models.^60^ Infection occurred despite the absence of commonly used bicarbonate^61^ or antibiotics^62^^,^^63^ pretreatment. We observed the highest levels of colonization with inoculation 6 h post-bowel prep, when the mucus layer was at its thinnest (Figures 3B–3F). These findings align with previous work in mice and humans showing that a compromised mucus layer enhances epithelial penetration and infection susceptibility.^27^^,^^64^^,^^65^^,^^66^ Our findings reveal that acute disruption of the gut environment alone is sufficient to reduce colonization resistance against enteric pathogens.

Normally, *Salmonella* Typhimurium needs a functioning flagellum to colonize the mammalian gastrointestinal tract,^61^ and previous studies have reported that motility disruption reduces fitness and invasiveness *in vitro* and *in vivo.*^67^^,^^68^^,^^69^ However, in the absence of mucus post-bowel prep, the non-motile *Salmonella* Typhimurium *ΔflhD* mutant was able to colonize and translocate to the mLN (Figure 4C). As the FlhD protein, a master regulator of flagellar biogenesis and motility, is involved in coordinating virulence,^70^^,^^71^ the ability of *Salmonella* Typhimurium *ΔflhD* to translocate suggests other virulence factors may facilitate translocation post-bowel prep. Specifically, type III secretion systems (T3SS) encoded by *Salmonella* pathogenicity islands 1 and 2 (SPI-1 and SPI-2) are independent of FlhD regulation and enable *Salmonella* Typhimurium to colonize, invade, and survive in the host without requiring motility.^72^^,^^73^ This finding expands our understanding of pathogen-host interactions in disrupted gut environments and suggests that non-motile pathogens may also be able to exploit these conditions to invade the gut mucosa.

Although mucus loss occurred throughout the mouse digestive tract, we observed pathology in *Salmonella* Typhimurium-infected cecal tissues but not in the colon (Figures 2E and 2F), unlike streptomycin-treated mice (Figures S2E and S2F). While a previous study suggested that the cecum is the preferred site of *Salmonella* Typhimurium infection due to incomplete mucus coverage,^30^ our findings suggest that the thinning of the cecal mucosa may further enhance tissue invasion at this location. Supporting this, hypertonic stimulation has been found to increase bacterial adhesion to host cells *in vitro.*^74^

Beyond the effects of mucus disruption, our mouse data suggest that microbiota depletion is a key driver of post-bowel prep pathogen susceptibility. After 24 h, osmolality and mucus had recovered, but luminal microbiota richness and SCFA levels remained depleted (Figures 3A–3D), and *Salmonella* Typhimurium was still able to colonize the gut (Figures 3E–3G). Higher SCFA levels, particularly butyrate, have been linked to lower *Salmonella enteritidis* burden in chickens through induction of host defense peptide gene expression,^75^ while depletion of butyrate-producing Clostridia increases aerobic expansion of *Salmonella* Typhimurium in mice.^76^ Given the observed reduction in intestinal butyrate levels post-bowel prep, it is possible that *Salmonella* Typhimurium experiences less virulence inhibition, contributing to its ability to invade tissue and translocate to extraintestinal organs (Figures 3E–3G). This aligns with a previous mechanistic study showing that microbiota-derived butyrate inactivates HilA, the master transcriptional activator of SPI-1, leading to reduced colonization in mice.^77^

Interestingly, our *in vitro* data suggest that PEG exposure alone can increase epithelial susceptibility to invasion but not intracellular expansion (Figures 5E–5J). This is consistent with prior reports showing that PEG-adjusted media decreases HT-29 cell viability and growth within a day of culturing,^78^ while higher osmolality media (mannitol-supplemented) disrupts tight junctions of Caco-2 intestinal epithelial cells.^79^ The observation that the cecal mucosa thins 6 h after bowel prep *in vivo* (Figure 1E), suggests that the treatment may directly weaken the barrier. On the bacterial side, osmotic stress was shown to increase intestinal epithelial cell line invasion across multiple *Salmonella* strains,^80^^,^^81^^,^^82^ as well as upregulation of virulence factors.^83^ Other enteric pathogens, including *Shigella* and enterohemorrhagic *Escherichia coli* O157:H7, also upregulate T3SS structural components and effector proteins under high-osmolality conditions.^84^^,^^85^ Thus, the combination of host epithelial disruption and pathogen hyperinvasiveness under osmotic stress likely acts synergistically, amplifying pathogen exploitation of host vulnerability.

Our mouse data show PEG treatment increases susceptibility to infection within hours, yet only a limited host response occurs during this acute window (Figures 5A and 5B), suggesting that epithelial cells are poorly equipped to counteract this perturbation within the timescale of maximal vulnerability. By contrast, other large-scale gut perturbations evoke stronger and faster transcriptional responses. For instance, *Salmonella* Typhimurium infection upregulates cytokine gene expression within 2.5 h in human intestinal organoids,^86^^,^^87^ and genes related to the inflammatory response within 8 h in the mouse colon.^88^ Interestingly, we found a coordinated enrichment of adaptive immune system GO terms in GSEA 6 h after bowel prep (Figures 5C and 5D), but this modest activation was insufficient to prevent *Salmonella* Typhimurium invasion and dissemination.

These findings show that bowel prep creates a short-lived but critical window in which both the host and invading pathogens are shifted toward a state of heightened susceptibility. While epithelial defenses are weakened and pathogens upregulate invasion pathways, an additional concern is that pathobionts already present within the microbiota—rather than only externally introduced pathogens like *Salmonella* Typhimurium—may also exploit this disruption. Importantly, we found that, unlike commensal bacteria, which display limited resilience to increased osmolality,^19^ IBD-associated pathobionts are highly tolerant to the osmotic perturbation induced by bowel prep and can expand under these conditions *in vitro* (Figure S7). However, in germ-free mice colonized with either IBD or healthy human microbiota, we detected no significant bacterial translocation or colitis two weeks after bowel prep (Figures S8A and S8B), suggesting that bowel prep in the absence of ongoing inflammation is not sufficient to exacerbate disease.

In contrast, when bowel prep was performed in the context of DSS-induced acute inflammation, colitis severity and pathology were significantly worsened in IBD microbiota-associated mice (Figures 6D and 6E). These mice also exhibited significantly increased extra-intestinal bacterial translocation relative to non-bowel prep controls (Figure 6F), mirroring the phenotype observed in *Salmonella* Typhimurium-challenged healthy mice. Sequencing of bacteria recovered from the mLN identified several IBD-associated pathobiont genera, including *Bacteroides*, *Lactobacillus*, and *Prevotella* (Figures 6G and S8H). In addition to higher live bacterial counts in extra-intestinal sites, a greater proportion of bowel-prepped mice exhibited bacterial translocation compared to controls; however, the number of distinct species translocated per mouse did not differ significantly between groups (Figure S8G). While severe DSS-induced tissue damage may permit many species to penetrate the epithelium,^64^^,^^89^ bowel prep may increase the likelihood of such translocation events occurring.

Notably, the IBD microbiota associated with milder colitis during DSS treatment produced greater histopathology scores and bacterial translocation post-bowel prep, suggesting that treatment impact is shaped by the specific microbiota context. These findings highlight the interplay between bowel prep, inflammation, and microbiota composition, suggesting that future clinical studies should investigate how these factors jointly influence the risk of symptom exacerbation following the procedure.^11^^,^^12^^,^^13^^,^^14^^,^^15^^,^^16^

In conclusion, this study establishes that, in both mouse and *in vitro* models, bowel prep rapidly alters the gut environment, increasing susceptibility to *Salmonella* Typhimurium and exacerbating inflammation in a colitis model. In addition, gut preparation promotes the translocation of intestinal bacteria from the gut to extraintestinal organs, such as nearby lymph nodes, the liver, and spleen. These findings suggest that gut preparation may have clinical implications that are not currently appreciated. Moreover, our findings suggest that other conditions that cause bouts of osmotic diarrhea, such as malabsorption and acute inflammation, may promote the growth and translocation of pathobionts that could further exacerbate disease states. While these findings provide important mechanistic insights, it is important to note that our results do not indicate a general risk to individuals undergoing clinically indicated bowel prep, but rather provide a model that highlights potential vulnerabilities that should be investigated in specific at-risk populations such as IBD patients.

### Limitations of the study

Differences in gut physiology and microbiota composition between humans and mice could limit the application of our findings to humans. Considerations such as transit time, size, and digestive tract physiology (particularly the cecum, where the greatest *Salmonella* pathology was observed) will have an impact on the susceptibility to pathogens in humans compared to mice. However, given the similarity of our model to clinical bowel prep and the support of our findings in an *in vitro* human cell line system, we believe our findings to be applicable. Future studies in humans should investigate the presence of specific pathobionts in biopsies. Importantly, previous findings already highlight the increased representation of potential Enterobacteriaceae pathobiont members such as *E. coli* and *Klebsiella* in IBD biopsies.^90^^,^^91^^,^^92^ However, the presence of these genera, alongside characterization of their pathobiont potential (e.g., the presence of toxin genes), needs to be correlated to health outcomes beyond the diagnosis at the time of colonoscopy. Still, given the technical challenges of measuring bacterial translocation in humans,^11^^,^^12^^,^^13^^,^^14^^,^^15^^,^^16^ this model may provide important insights into which bacteria should be monitored in patients requiring more frequent colonoscopies, including those with IBD.

Similarly, differences between our *in vitro* model and the actual human gut may limit application of findings from this study system. However, in the bowel prep system, due to the much reduced microbiota and mucus layer following the laxative treatment, this model is particularly apt to model pathogen infection in the context of PEG exposure. For instance, HT-29 cultures are an established model for studying pathogen interactions with the intestinal epithelium.^33^^,^^34^ Although they contain a small subset of mucus-secreting cells,^93^ they produce little mucus overall, reflecting the transient mucus-depleted state of the epithelium observed *in vivo* after bowel prep.

Taken together, while the study systems used here have inherent limitations, their ability to mimic key aspects of bowel prep and pathogen susceptibility supports their utility in exploring the mechanisms underlying microbial translocation and infection.

## Resource availability

### Lead contact

Further information and requests for resources and reagents should be directed to and will be fulfilled by the lead contact, Carolina Tropini (carolina.tropini@ubc.ca).

### Materials availability

This study did not generate new unique reagents.

### Data and code availability

• All data are available at https://doi.org/10.5683/SP3/DGHLV4. Transcriptomics and 16S rRNAseq raw reads are available on NCBI; see Deposited Data in the key resources table.
• All original code is available at https://doi.org/10.5683/SP3/DGHLV4.
• Any additional information required to reanalyze the data reported in this paper is available from the lead contact upon reasonable request.

## Acknowledgments

The authors acknowledge that the land we performed this research on is the traditional, ancestral, and unceded territory of the xwməθkwəỳəm (Musqueam) Nation. The land our laboratory is situated on has always been a place of learning for the Musqueam people, who for millennia have passed on their culture, history, and traditions from one generation to the next on this site. We encourage others to learn more about the native lands in which they live and work at https://native-land.ca/.

The authors thank Denise Monack, Aaron Dhanda, Christopher Lee, and members of the Tropini Lab for useful conversations and insight; Michael Hunter for critically reading the manuscript; Sophie Cotton for assisting in preliminary pilot experiments; and Angele Arrieta for managing lab processes. We acknowledge the UBC Center for Disease Modeling and Natalia Carranza Garcia for support with animal work. We thank Ben Swenor and William Bralower for assistance with gut-on-a-chip experiments, Kunho Choi and the Vallance Lab for providing reagents, and Wanyin Deng and the Finlay Lab for providing *Salmonella* Typhimurium strains. This study received support from the Life Sciences Institute Biofactorial High-Throughput Biology Core, supported by the UBC Global Research Excellence Biological Resilience Initiative. We thank Harper Health & Science Communications, LLC, for editorial support of the initial manuscript submission. The authors acknowledge support from the 10.13039/100007631Canadian Institute for Advanced Research/Humans and the Microbiome (FL-001253 Appt 3362), 10.13039/501100000245Michael Smith Foundation for Health Research Scholar Award (18239), Canada Foundation for Innovation/Infrastructure Operating Fund (38277), Canada Tier 2 Research Chair, Quantitative Microbiota Biology for Health Applications (CRC-2022-00036), Canadian Institutes of Health Research Project grant (PJT 191743), 10.13039/501100007658Crohn's and Colitis Canada/Grants In Aid of Research (625155) (to C.T.); Paul Allen Foundation/Allen Distinguished Investigators (ADI) Program (12935, to C.T. and S.R.S.); The W. Garfield Weston Foundation (Weston Family Microbiome Initiative, to C.T.); and the CH.I.L.D Foundation Chair in Pediatric Gastroenterology (to B.A.V.).

## Author contributions

C.A.C.: conceptualization, methodology, formal analysis, investigation, data curation, writing – original draft, writing – review & editing, visualization; I.P.: conceptualization, methodology, formal analysis, investigation, data curation, writing – original draft, writing – review & editing, visualization; B.D.D.: conceptualization, methodology, formal analysis, investigation, data curation, writing – original draft, writing – review & editing, visualization; G.M.: methodology, formal analysis, investigation, writing - review & editing, visualization; A.S.: investigation, formal analysis; C.S.: investigation; J.Y.H.: formal analysis; A.D.P.: investigation; D.T.: investigation; D.M.P.: investigation; T.F.: investigation; K.M.N.: conceptualization, methodology, investigation, writing - review & editing, supervision; S.R.S.: conceptualization, resources, supervision; M.G.S.: resources, supervision; B.A.V.: conceptualization, resources; C.T.: conceptualization, resources, writing – original draft, writing– review & editing, formal analysis, supervision, project administration, funding acquisition.

## Declaration of interests

The authors declare no competing interests.

## STAR★Methods

### Key resources table

**Table:** 

REAGENT or RESOURCE	SOURCE	IDENTIFIER
**Bacterial and virus strains**		

*Salmonella enterica* serovar Typhimurium SL1344	Gift from Finlay Lab	N/A
*Salmonella enterica* serovar Typhimurium SL1344 flhD-	Gift from Finlay Lab	N/A
Constitutive mCherry-expressing *Salmonella enterica* serovar Typhimurium SL1344	Knodler et al.^38^	N/A

**Biological samples**		

Human fecal samples from patients with ulcerative colitis and healthy controls	Sinha Lab	N/A
Potential pathobiont Enterobacteriaceae from patients with IBD library	Surette Lab	N/A

**Chemicals, peptides, and recombinant proteins**		

Polyethylene Glycol 3350	RestoraLAX	N/A
Methanol (Certified ACS)	Fisher Chemical	A412P-4
Chloroform (Ethanol as Preservative/Certified ACS)	Fisher Chemical	C298-500
Acetic Acid, Glacial (Certified ACS)	Fisher Chemical	X3P-1GAL
DAPI	Sigma Aldrich	D9542-5MG
WGA	Vector Laboratories	RL-1022
UEA-1	Vector Laboratories	FL-1061
Hematoxylin Solution, Gill No. 2	Sigma Aldrich	GHS216
Hydrochloric Acid Concentrate, 10N ACS (Certified)	Fisher Chemical	SA49
Ethyl Alcohol Anhydrous, USP	Greenfield Global	P016EAAN
Sodium Bicarbonate (Fine White Powder)	Fisher BioReagents	BP328-500
Magnesium Sulfate Heptahydrate	Fisher BioReagents	BP213-1
Eosin Y Solution, Alcoholic	Sigma Aldrich	HT110116
Permount™ Mounting Medium	Fisher Chemical	SP15-100
Phosphoric Acid, Certified, 25.0% (v/v) ±0.5% (v/v)	LabChem	LC186551
LB Broth, Miller	Fisher BioReagents	BP1426-500
Streptomycin sulfate salt	Sigma Aldrich	S6501-50G
Columbia Broth	BD DIFCO	294420
Sheep Blood (Defibrinated)	Dalynn Biologicals	HS30-500
Hemin Chloride	MilliporeSigma	37415GM
Vitamin K{1}	Thermo Scientific Chemicals	AAL1057506
DMEM, high glucose	Sigma-Aldrich	D6429
Fetal Bovine Serum, Regular	Corning	35-077-CV
Penicillin-Streptomycin	Sigma-Aldrich	P4333
GlutaMAX™ Supplement	Thermo Fisher	35050061
TrypLE	Thermo Fisher	12605010
Non-essential Amino Acid Solution	Sigma-Aldrich	M7145
Gentamicin	Sigma-Aldrich	G3632-5G
Triton X-100	MilliporeSigma	TX1568-1
ReadyProbes™ Reagent F-Actin Phalloidin Conjugates	Invitrogen	R37110
Normal Donkey Serum	Jackson ImmunoResearch	017-000-121
Bovine Serum Albumin	Sigma-Aldrich	A7906
VECTASHIELD®	BioLynx	VECTH1000
Paraformaldehyde 16% Aqueous Solution EM Grade	Electron Microscopy Sciences	15710-S
Collagen IV	Sigma-Aldrich	C5533
Fibronectin	Corning	CLS356008
Corning™ Matrigel™ Matrix	Corning	CB-40234
Endothelial Cell Growth Medium MV 2	Sigma-Aldrich	C-22121
Attachment Factor	Cell Systems	4Z0-210
HBSS, calcium, magnesium	Thermo Fisher	14025092
Fluorescein isothiocyanate-dextran	Sigma-Aldrich	FD4
DPBS, no calcium, no magnesium	Gibco	14190144
Dextran sodium sulfate	ThermoFisher	J6360622

**Critical commercial assays**		

RNeasy Mini Kit	Qiagen	74104
S1 Chip	Emulate	N/A

**Deposited Data**		

16S rRNA seq – humanised IBD mouse microbiota (Figures 6 and S8)	This work	NCBI BioProject: PRJNA1348478
16S rRNA seq – conventional mice microbiota (Figures 1, S1, 3, and S3)	This work	NBCI BioProject: PRJNA1348202
*In vivo* transcriptomics	This work	NCBI BioProject: PRJNA1348472

**Experimental models: Cell lines**		

Caco-2 Cells	ATCC	HTB-37; RRID: CVCL_0025
HT-29 Cells	ATCC	HTB-38; RRID: CVCL_0320
Human Intestinal Microvascular Endothelial Cells	Cell Systems	ACBRI 666

**Experimental models: Organisms/strains**		

Mouse: C57BL/6J	Tropini Lab (originally Jackson Laboratories)	RRID: IMSR_JAX:000664
Mouse: Swiss Webster	Tropini Lab (originally Taconic)	RRID: IMSR_TAC:SW

**Oligonucleotides**		

16S V4 region 515F/926R primers (Forward primer: GTGYCAGCMGCCGCGGTAA-3'. Reverse primer: CCGYCAATTYMTTTRAGTTT-3')	IDT	N/A
16S V4 region 515F/806R primers (Forward primer: GTGYCAGCMGCC GCGGTAA-3′, Reverse primer: GGACTACNVGGGTWTCTAAT-3′)	IDT	N/A

**Software and algorithms**		

Custom code for this project	This work	https://doi.org/10.5683/SP3/DGHLV4.
ImageJ (version 2.16.0/1.54p)	https://imagej.net/ij/	N/A
FlowJo (version 10.10.0)	https://www.flowjo.com/	N/A
BacSpace	Earle et al.^94^	N/A
QIIME2 (version 2023.9)	Bolyen et al.^95^	N/A
DADA2	Callahan et al.^96^	N/A
Tidyverse (version 2.0.0)	Wickham et al.^97^	N/A
ggplot2 (version 3.5.21)	Hadley, 2016^98^	N/A
Fastp (version 0.23.4)	Chen et al.^99^	N/A
STAR 2.7	Dobin et al.^100^	N/A
DESeq2 (version 1.42.1)	Love et al.^101^	N/A
EnhancedVolcano (version 1.20.0)	Blighe et al.^102^	N/A
Pheatmap (version 1.0.13)	Kolde.^103^	N/A
clusterProfiler (version 4.10.1)	Wu et al.^104^	N/A
org.Mm.e.g.,.db (version 3.18.0)	Carlson.^105^	N/A
ggridges (version 0.5.6)	Wilke.^106^	N/A
UpSetR (version 1.4.0)	Gehlenborg.^107^	N/A

### Experimental model and study participant details

#### Bacterial strains

This study utilized naturally streptomycin resistant *Salmonella enterica* serovar Typhimurium SL1344 as a model enteric pathogen, as well as *Salmonella* Typhimurium *ΔflhD* from the Finlay Lab (UBC) and mCherry-*Salmonella* Typhimurium. These strains were grown in Luria-Bertani Miller medium supplemented with streptomycin (100 μg/mL).

#### Animal handling and ethics

All animal experiments were conducted in accordance with the ethical guidelines of the University of British Columbia’s (UBC) animal care procedures, following protocol number A23-0115 approved by the Animal Care Committee. C57BL/6J male and female mice between 8 and 12 weeks of age were used and were provided with an autoclaved standard diet (Purina LabDiet 5K67). Mice were randomly allocated to experimental groups with equal numbers of each sex. As we did not observe sex differences, male and female mice were analyzed together in all experiments. Experimental groups that underwent oral gavage were monitored at 1 h and 24 h post-procedure. Daily health checks for mortality after infection were performed, and mice were euthanized if they lost more than 20% of their body weight or displayed signs of distress. Euthanasia at indicated tissue-collection time points was performed using carbon dioxide asphyxiation followed by cervical dislocation.

#### Mammalian cell culture

HT-29 (human, female) and Caco-2 (human, male) cells were obtained from the American Type Culture Collection (ATCC). Cells were cultured individually in Dulbecco’s Modified Eagle Medium (DMEM) high glucose (Sigma-Aldrich) supplemented with 10% (v/v) heat-inactivated fetal bovine serum (FBS, Corning), 1% (v/v) Penicillin-Streptomycin (Sigma-Aldrich), 1% (v/v) GlutaMAX supplement (Thermo Fisher), and 1% (v/v) non-essential amino acid (NEAA, Sigma-Aldrich) solution in T75 flasks (Corning). Cells were grown in humidified incubators at 37°C and 5% CO_2_. Media was replaced every other day and cells were passaged once they reached 60–70% confluency using 3 mL of TrypLE (Thermo Fisher) in a 1:10 split ratio.

Human Intestinal Microvascular Endothelial Cells (HIMECs, sex not specified) were cultured with complete endothelial cell growth medium (Sigma-Aldrich) and 1% (v/v) Penicillin-Streptomycin (Sigma-Aldrich) in humidified incubators at 37°C and 5% CO_2_. Frozen cells at passage 7 were thawed a week before experiment and seeded into a T75 flask (Corning) coated with 5 mL of Attachment Factor (Cell Systems). Media was changed every other day and cells were passaged once at 80% confluency using 3 mL of TrypLE by seeding 1 × 10^5^ cells into an Attachment Factor-coated T75 flask.

Cell lines were not authenticated.

#### Human fecal samples

All studies were approved by the Stanford University Institutional Review Board (IRB). Fecal samples were collected from patients with confirmed ulcerative colitis (UC) and from healthy controls. Samples were collected by patients at home in BIOME-Preserve tubes according to manufacturer instructions and returned to the research team within 24 h, then snap-frozen with dry ice and stored at −80°C until use.

Each *in vivo* humanised mouse experiment utilising human fecal samples for microbiota inoculation was fully separate, meaning all mice within an experiment were allocated the same microbiota. One healthy human sample obtained from a 48-year-old female patient was used for the humanised control mice (hHealthy: *n* = 9 [Figure S8B]). Samples collected from 62- and 51-year-old male patients with ulcerative colitis were used for the humanised IBD mice (hIBD1: *n* = 10 [Figures 6 and S7], *n* = 9 [Figure S8B]; hIBD2: *n* = 15 [Figures 6 and S7]). Inocula for gavage were prepared in an anaerobic chamber, where 5 g of feces from stool samples was resuspended in 10 mL of sterile and pre-reduced PBS and centrifuged at 4000 RPM for 5 min.

### Method details

#### Bowel prep mouse model

Bowel prep solution was prepared by dissolving 42.5% (w/v) polyethylene glycol (PEG) (commercially branded Restoralax) in ddH_2_O and filter sterilized. Food was withdrawn from all cages 1 h before the bowel prep procedure. Mice were orally gavaged with 200 μL of bowel prep solution every 20 min a total of 4 times. Vehicle-treated mice were orally gavaged with 200 μL of water following the same dosing time as bowel prep-treated mice. Food and water were readministered after the procedure.

This concentration and delivery protocol were selected to reflect PEG dosing used in human bowel prep regimens, which typically involve administration of up to 420 g of PEG over a short time frame, corresponding to an estimated 4.9–10.5 g PEG/kg body weight depending on patient size. In our model, mice received a total of ∼0.34 g PEG (administered as 200 μL every 20 min for four doses), corresponding to ∼7.2–13.6 g PEG/kg, a dose range physiologically comparable to human protocols when adjusted for mouse body weight and intestinal clearance. To account for potential hydration differences introduced by the vehicle, mice were monitored for signs of dehydration and provided with *ad libitum* access to water immediately following the procedure.

We additionally evaluated the impact of high dose osmotic laxatives on the gut environment and the gut microbiota at 6, 24, 48, and 72 h post-bowel prep compared to untreated controls sacrificed at the 6-h timepoint (baseline).

#### Cecal osmolality and pH measurements

Mice were sacrificed, and cecal contents were collected and placed on ice. The osmolality of the cecal contents was directly measured using an Advanced Instruments Osmo1 Single-Sample Micro-Osmometer (Fisher Scientific). pH was measured with a calibrated micro pH probe (Orion PerpHecT ROSS Combination pH Micro Electrode).

#### Tissue collection

Sterile tools were used to collect the lower lobe of the liver, mesenteric lymph nodes, and the spleen. The tissues were placed in pre-weighed sterile 2 mL tubes containing 200 μL of PBS and were put on ice. Cecal contents were collected, and half of the contents were flash-frozen on dry ice, while the other half was placed on ice for downstream analysis. The ileum, cecum, and colon tissues were collected in histology cassettes and immediately fixed in methacarn solution (60% dry methanol [Fisher Chemical], 30% chloroform [Fisher Chemical], 10% glacial acetic acid [Fisher Chemical]) for 7 to 14 days. The samples were then washed twice in methanol for 30 min, twice in 100% ethanol (Sigma) for 20 min, and twice in xylenes (Fisher Chemical) for 15 min. The tissues were coated and incubated in paraffin at 60°C for 2 h and then dried at room temperature. Paraffin blocks were cut into 4 μm sections and mounted on slides by the British Columbia Children’s Hospital Research Institute’s Histology Core.^108^

#### Lectin staining of the GIT

Paraffin was removed from sectioned slides through incubation at 60°C for 10 min, followed by 2x 10-min incubations in pre-warmed xylenes at 60°C. Slides were then incubated in 99.5% ethanol for 5 min, then left to dry and circled with a PAP (liquid blocker) pen (Fisher Scientific).^108^ DAPI (10 μg/mL, Sigma Aldrich), UEA-1 (Fluorescein-labeled Ulex Europaeus Agglutinin I, 40 μg/mL, Vector Laboratories), and WGA (Rhodamine Red-X-labeled Wheat Germ Agglutinin, 30 μg/mL, Vector Laboratories) were applied to fully cover the samples (approximately 250 μL) and incubated in the dark at 4°C for 45 min. The slides were then washed in PBS 3 times. Sections were left to dry for 5 min, and ProLong Gold Antifade Mountant, (Invitrogen), was applied, followed by a #1.5 glass coverslip.^108^ Images were collected using a Zeiss LSM 900 confocal microscope at 100x (Figures 1 and S1) or 20x (Figures 3 and S3) magnification with the ZEN 2020 software. Mucus thickness was quantified using the analysis platform BacSpace.^94^

#### Confocal image selection and mucus quantification

Confocal images of longitudinal sections of the cecal tip and distal colon were collected for 5–8 mice per treatment group. For each tissue, three representative sites with ≥ 500 μm continuous epithelium were imaged per mouse. Whenever possible we used sections that contained intestinal contents; however, in the case of bowel prep the contents were purposely evacuated by the procedure. These sites were selected for continuous epithelium and the presence of intact intestinal contents, where possible.

The contour defining the boundary of the epithelium and the mucus layer were identified and defined using the BacSpace MATLAB software.^94^ A mucus layer was defined as having minimum thickness of 5 μm and no non-bacterial gut contents (e.g., large fiber pieces). Using a custom MATLAB Script, mucus-covered area was defined as the region(s) between these curves. Percentage mucus coverage was calculated as the total length of the mucus divided by the total length of the epithelium. Average mucus thickness was calculated as the total covered by mucus divided by the total length of the epithelium. Quantitative analyses were performed on each image, and the mean value from the three images was calculated to represent a single data point per mouse. Differences in average thickness between treatment and control groups (Figure 1) or between timepoints post-bowel prep (Figures 3 and S3) were assessed using a one-way ANOVA.

#### Hematoxylin & eosin tissue histology

Paraffin was melted from the section slides by heating the slides in coplin jars at 60°C for 10 min. Slides were then incubated in the coplin jars in 60°C xylenes for 3 min twice followed by 2 incubations in 100% EtOH for 2 min each, 95% EtOH for 2 min, and in ddH20 for 2 min.^109^ Next, the hematoxylin stain was performed by filling the coplin jars with Hematoxylin Solution, Gill’s No. 2 (Sigma Aldrich, GHS216), and incubating for 3 min, then in ddH_2_O for 1 min, differentiator solution (0.3% v/v 10N HCl [Fisher Chemical], 70% v/v EtOH [Sigma] in ddH2O)) for 30 s, ddH20 for 1 min, and blueing reagent (0.2% w/v NaHCO_3_ [Fisher BioReagents], 4.1% w/v MgSO_4_ᐧ6H_2_O [Fisher BioReagents] in ddH2O)) for 1 min. For the Eosin stain, slides remained in the coplin jar and were filled with 95% EtOH for 1 min followed by Eosin Y solution (Sigma Aldrich), for 45 s. The stain was followed by washes in 95% EtOH for 1 min, in 100% EtOH for 1 min 3 times, and in xylenes for 2 min twice. Stained slides were mounted using Permount (Fisher Chemical), a xylene-based mounting medium, and allowed to solidify for at least 24 h.^109^ H&E images were acquired using the 3D HISTECH Slide Scanner at 37x (Figures 1E and 2E) or the ZEISS Digital Slide Scanner Axioscan 7 at 20x (Figures 4C and 6D).

#### *Salmonella* Typhimurium infection pathology scoring

H&E-stained sections of the cecum and distal colon were assessed for pathology in the lumen, epithelium, mucosa, and submucosa of the gut. Luminal pathology was scored based on the presence of necrotic epithelial cells and polymorphonuclear neutrophils (PMNs) (0 = none, 1 = scant, 2 = moderate, 3 = dense). The epithelium was scored for desquamation (0 = no change, 1 = limited shedding, 2 = moderate shedding per lesion), regenerative change (0 = none, 1 = mild, 2 = moderate, 3 = severe), ulceration (1 = epithelial ulceration), and PMNs in the epithelium (1 = PMNs present). The mucosa was scored based on crypt abscesses (0 = none, 1 = mild, 2 = moderate, 3 = severe) and the presence of mucin plugs and granulation tissue (1 = present). Lastly, the submucosa was scored for edema, mononuclear cell infiltration, and PMN infiltration (0 = no change, 1 = mild, 2 = moderate, 3 = severe). Scores for each location across the image were summed together, with the maximum pathological score being 24 per image. Two images of 1000 μm continuous mucosa were taken for each mouse, and were scored by two blinded scorers. Scores were averaged for each mouse.

#### Mucosa thickness measurements

20× images of H&E-stained tissues were exported in TIFF format from SlideViewer. TIFF images were converted from RGB to 8-bit format in FIJI, and pixel values were inverted so that tissues appeared light against a dark background. BacSpace was used to create two masks; one mask outlined the submucosal edge of the mucosa, and the other mask outlined the luminal edge of the epithelium. Both masks were applied to the original image so that only the mucosa and epithelium were non-zero values. BacSpace was then used on the masked images to straighten the image relative to the luminal edge of the epithelium. The luminal edge of the epithelium in the straightened image was detected as the greatest decrease in signal along the x axis of the straightened image at each point along the epithelium. The mean thickness was quantified for each image, and the mean thicknesses of two images from the cecum and two images from the distal colon from three different mice per treatment (total = 6 images per treatment per organ) were compared using a Wilcoxon Rank-Sum Test.

#### Quantification of gut permeability

Intestinal permeability was assessed in mice following bowel prep using a FITC-dextran assay.^110^^,^^111^ Mice with a conventional microbiome received bowel prep (*n* = 12) or a vehicle control (*n* = 12), then continued to be fasted for an additional 2 h before receiving 150 μL of 80 mg/mL fluorescein isothiocyanate (FITC)–dextran (4 kDa, Sigma-Aldrich) in PBS by oral gavage. Food and water were readministered after the FITC-dextran gavage. Mice were sacrificed 4 h following FITC-dextran administration, and serum was collected by cardiac puncture. The fluorescence of FITC in serum was measured in duplicate using emission wavelength of 485 nm and emission wavelength of 535 nm using a Biotek Synergy H1 plate reader. A standard curve was generated using serial dilutions of FITC-dextran, and serum from untreated mice was used to account for background fluorescence.

#### Quantification of bacterial levels in feces

1 μL of feces was diluted in 200 μL of sterile PBS. The solution was serially diluted at 1:10 down a 96-well plate. Next, 5 μL fecal dilutions were spot-plated on both LB-streptomycin agar plates and Columbia Blood agar plates. Plates were incubated aerobically overnight at 37°C. Single colonies were counted from the highest possible dilution and back calculated to determine the absolute microbial abundance.

#### DNA extraction, library preparation, 16S rRNA sequencing and analysis

DNA was extracted from fecal pellets and cecal contents using the DNeasy 96 PowerSoil Pro QIAcube HT Kit (Qiagen Inc., Valencia, CA) according to the manufacturer’s instructions.

For all 16S rRNA runs except for the *in vitro* IBD (Figures S7A–S7D), 16S rRNA library preparation was conducted at the Biofactorial High-Throughput Biology (Bio!) Facility at the University of British Columbia. Amplification of the V4 region was performed with 515F/926R primers (515F, 5′- GTGYCAGCMGCCGCGGTAA-3’; 926R, 5′-CCGYCAATTYMTTTRAGTTT-3′). Pooled libraries were then submitted to the Bio! facility, where sequencing was performed on the Illumina MiSeq platform with v2 2 × 300 bp paired-end read chemistry. For the *in vitro* IBD samples, 16S library preparation was performed by Gut4Health as previously described.^112^ Amplicons of the V4 region of the 16S rRNA were generated using KAPA HiFi HotStart Real-time PCR Master Mix (Roche) and barcode primers 515F: GTGYCAGCMGCCGCGGTAA and 806R: GGACTACNVGGGTWTCTAAT. Purified PCR libraries were normalized and pooled with the SequalPrepTM normalization plate (Applied Biosystems). Library concentrations were confirmed using the QubitTM dsDNA high sensitivity assay kit (Invitrogen) and KAPA Library Quantification Kit (Roche). The purified pooled libraries were then submitted to UBC’s Bioinformatics and Sequencing Consortium (SBC). Paired end read sequencing was carried out either on the Illumina MiSeq v3 platform with 2 × 300 bp paired end-read chemistry or the NextSeq 600 cycle P1 with 2 × 301 bp. To ensure DNA quality and quantity, an Agilent high sensitivity DNA kit (Agilent) was employed on an Agilent 2100 Bioanalyzer.

Read quality was assessed by running FASTQC^113^ on the generated FASTQ files. Reads were then imported into QIIME2-2023.9 for subsequent analyses.^95^ DADA2 (via q2-dada2) was used to denoise and quality filter the data; then reads were trimmed to remove primer sequences while maintaining mean Phred quality scores >Q30.^96^ Using the QIIME classification plugin (q2-feature-classifier), amplicon sequence variants (ASVs) were classified via a naive Bayes machine-learning taxonomic classifier against the SILVA 138 99% identity reference sequence database.^114^ Multiple sequence alignment and phylogenetic tree generation was performed using MAFFT (via q2-alignment) and FastTree2 respectively (via q2-phylogeny).^115^^,^^116^ Plotting was conducted using R v4.2.2.^117^ Tidyverse^97^ and ggplot2^118^ packages were used for data visualization. The packages phyloseq,^119^ ggpubr,^120^ and vegan^121^ were used for sample rarefaction, calculation, and visualization of alpha and beta diversity metrics.

#### SCFA extraction from cecal contents

SCFAs were extracted from 40 to 100 mg of flash-frozen cecal contents. The samples were homogenized with 0.8 mL of 25% phosphoric acid (LabChem) and centrifuged at 15,000 × g for 10 min at 4°C. The supernatant was removed from all samples and centrifuged again. Subsequently, 800 μL of the supernatant was filtered through a 0.45 μm filter (Fisher) and mixed with 0.2 mL of the internal standard solution containing 24.5 mmol/L isocaproic acid in a GC vial (12 × 32mm, Thermo Scientific). SCFAs were quantified by gas chromatography-mass spectrometry by the AFNS Chromatography Facility at the University of Alberta, as previously described.^122^

#### *Salmonella* Typhimurium culture and inoculum preparation

Glycerol stocks of the naturally streptomycin resistant *Salmonella enterica* serovar Typhimurium SL1344 strains (*Salmonella* Typhimurium [WT] and *Salmonella* Typhimurium *ΔflhD*, mCherry-*Salmonella* Typhimurium) were streaked onto Luria-Bertani Miller (LB) 1.5% agar plates supplemented with streptomycin (100 μg/mL). Following incubation at 37°C for 24 h without agitation, a single *Salmonella* Typhimurium colony was selected from the agar plate and transferred to 5 mL of LB liquid broth containing 100 μg/mL streptomycin. The culture was then aerobically incubated at 37°C with shaking at 200 rpm for 17 h. Subsequently, the cultures were serially diluted in PBS to achieve the desired inoculant bacterial counts.

#### *Salmonella* Typhimurium osmolality growth measurements

Wildtype *Salmonella* Typhimurium or *Salmonella* Typhimurium *ΔflhD* were diluted 1:100 from overnight LB cultures in fresh LB media adjusted to ∼400, 800, 1200, and 1800 mOsm/Kg using PEG (Restoralax). Growth curves were obtained using a Biotek Synergy H1 plate reader at 37°C for 24 h aerobically. The plates were shaken orbitally every 15 min following the collection of OD600 measurements. Background absorbance subtraction was performed from non-inoculated wells. The growth rates of the strains were determined using a previously published MATLAB package.^19^

#### Streptomycin *Salmonella* Typhimurium infection model

Conventional mice were fasted for 4 h before being treated with 100 μL of 200 mg/mL streptomycin (Sigma-Aldrich, S6501-50G) by oral gavage.^29^ 24 h later, mice were fasted for 3 h and infected by oral gavage with 100 μL of 10^6^ CFU *Salmonella* Typhimurium. Fecal samples were collected daily for three days after *Salmonella* Typhimurium infection and spot-plated on streptomycin-LB agar plates.

#### Bowel prep *Salmonella* Typhimurium infection model

Conventional mice underwent bowel prep as described previously and received oral gavage with 100 μL of 10^6^ CFU of wildtype *Salmonella* Typhimurium or *Salmonella* Typhimurium *ΔflhD* 6 h after the bowel prep. To assess dose-dependent *Salmonella* Typhimurium colonization, mice were infected with 10^2^, 10^3^, 10^5^, 10^6^, and 10^9^ CFU post-bowel prep. To explore the susceptibility to *Salmonella* Typhimurium infection following bowel prep over time, mice were infected with 10^6^ CFU of *Salmonella* Typhimurium 6, 24, and 48 h post-bowel prep. Fecal samples were collected daily for three days after *Salmonella* Typhimurium infection and spot-plated on Columbia Blood agar plates (per liter: 35 g Columbia Broth, 5% Sheep Blood, hemin, and vitamin K) to determine total absolute bacterial abundance and streptomycin-LB agar plates to determine absolute abundance of *Salmonella* Typhimurium.

#### RNA extraction, sequencing and analysis

Cecal tip tissue was collected from conventional CL57/6J mice treated with bowel prep and sacrificed 6 h later alongside an untreated baseline group. RNA was extracted from cecal tip tissue using the RNeasy Mini Kit (Qiagen) according to the manufacturer’s instructions. The extracted RNA was sent to the Sequencing and Bioinformatics Consortium at UBC, where a sequencing library was prepared using a standard Illumina Stranded mRNA kit (Illumina) to generate 150 bp pair-end reads. Raw reads were assessed for nucleotide quality (terminal bases below PHRED quality score 20 were removed), trimmed to remove adaptor sequences, and merged using Fastp v. 0.23.4.^99^ The reads were then aligned to the mouse genome GRCm39 using the STAR 2.7 software^100^ with --quantMode GeneCounts, and the gene counts obtained were imported into R for downstream analyses.

Raw gene counts were analyzed using DESeq2.^101^ Volcano plots of genes with adjusted *p*-value (Benjamin-Hochberg corrected) less than 0.05 and log_2_ fold change greater than 1.5 were plotted using the EnhancedVolcano package.^102^ Heatmaps of 40 select genes of interest and the top 50 most variable genes by absolute *Z* score were plotted with the pheatmap package.^103^ Gene set enrichment analysis (GSEA) was performed using the clusterProfiler package.^104^ Genes were ranked by multiplying the sign of log_2_ fold change with the -log_10_(*p*-value) obtained from the DESeq2 results. Gene symbols were converted to Entrez IDs using the org.Mm.e.g.,.db package^105^ and enrichment analysis was performed using the Gene Onology (GO) biological process database including gene sets between 10 and 400. The normalized enrichment score (NES) of GO terms with a *p*-value below 0.05 was visualized using the ggplot2 package.^118^ In addition, the log_2_ fold change of genes within the leading edge subset of each enriched GO term was extracted and plotted using the ggridges^106^ and upsetR^107^ packages.

#### HT-29 monolayer PEG exposure, invasion, and gentamicin protection models

Circular glass coverslips (Fisherbrand) in a 24-well plate (Corning) were coated with 100 μL of Bovine Collagen I (Corning) diluted 1:60 in 0.01 N hydrochloric acid (Fisher Chemical). HT-29 cells between passages 18–25 were detached with TrypLE (Thermo Fisher) and resuspended to 2 × 10^6^ cells/mL, then 100 μL of cells were seeded to each coated coverslip along with 400 μL of supplemented DMEM media. Cells were cultured for four days before *Salmonella* Typhimurium infection, and media were changed every second day. The day before infection, media were exchanged with antibiotic-free supplemented DMEM.

For PEG dosage experiments, basal DMEM media adjusted to 600, 900, or 1200 mOsm/kg with PEG was added to each well at 24 h, 3 h, and 1 h prior to infection. Monolayers were infected with late-log phase *Salmonella* Typhimurium at a multiplicity of infection (MOI) of 50. After 30 min, all wells were treated with 100 μL/mL gentamicin (Sigma Aldrich) in basal DMEM for 1 h. To obtain counts for intracellular invasion, monolayers were lysed with 1% Triton X-100 (MilliporeSigma) and 1:10 serial dilutions were plated on LB-strep plates.

For flow cytometry and microscopy experiments, basal DMEM media adjusted to 600 mOsm/kg with PEG was added to each well 3 h before infection. Monolayers were infected with late-log phase mCherry-expressing *Salmonella* Typhimurium^35^ at an MOI of 50. After 1 h, all wells were treated with 10 μL/mL gentamicin in basal DMEM for 2 h.

#### Flow cytometry of HT-29 monolayers

Following infection endpoint, cells were detached with TrypLE (Thermo Fisher) and pelleted at 5,000 × g for 5 min, then fixed in 2% paraformaldehyde and resuspended in PBS to be analyzed on a CytoFLEX LX flow cytometer. mCherry signal was excited with the 561 nm yellow laser and detected using a 610/20 nm bandpass filter. Cells and singlets were gated, and at least 25,000 events were recorded from the singlets gate for each sample at medium speed (30 μL/min). FCS files were imported into FlowJo (v10.10.0) and an mCherry-positive gate was defined using negative control samples, with thresholds set to include <1% of events from the control populations. All events within the singlets gate for each sample were exported and visualized in R using the ggplot2 package.^118^

#### Staining and imaging of HT-29 monolayers

Monolayers at infection endpoint were fixed with 4% paraformaldehyde for 15 min. Cells were permeabilized with 0.2% Triton X-100 and blocked with 5% normal donkey serum (NDS) and 1% bovine serum albumin (BSA) in PBS. Cells were stained with DAPI (Sigma Aldrich) and Alexa-Fluor 488-conjugated phalloidin (Fisher) and mounted to slides using Vectashield (BioLynx). Cells were imaged using a Zeiss LSM 900 confocal microscope and analyzed using ImageJ Fiji (v. 2.16.0/1.54p). 50 Z-stacks with 0.5 μm intervals were taken of each image to ensure coverage of the entire cell, and infected cells and mCherry-*Salmonella* Typhimurium were blindly counted from max intensity projections of images. Four fields of view (FOVs) from each of three technical replicates of each condition were imaged and analyzed, across three independent experiments.

#### Gut-on-a-chip culture

S1 chips (Emulate) were surface activated according to the manufacturer’s protocol. Chips were then coated overnight in humidified incubators at 37°C and 5% CO_2_ with 200 μg/mL Collagen IV (Sigma-Aldrich) and 100 μg/mL Matrigel (Corning) in the apical channel, and 200 μg/mL Collagen IV and 30 μg/mL Fibronectin (Corning) in the basolateral channel, prepared in sterile cell-culture grade water. The following day, HIMECs at passage 8 were detached with TrypLE then resuspended to 8 × 10^6^ cells/mL and 15 μL was seeded to the basolateral channel. Chips were flipped upside down and incubated for 1 h to allow HIMECs to adhere. After flipping chips back, 4 parts of Caco-2 at passage 17 and 1 part of HT-29 at passage 16 detached with TrypLE were combined and resuspended to 7.5 × 10^6^ cells/mL, and 35 μL was seeded to the apical channel. Cells were allowed to adhere overnight in the incubator, before connecting chips to continuous flow (60 μL/h) and mechanical deformation (10%, 0.15 Hz) and grown for 3 additional days before switching to antibiotic-free DMEM high glucose with supplements in both channels.

#### Chip bowel prep and *Salmonella* Typhimurium translocation model

Bowel prep solution was prepared by dissolving 0.05 M polyethylene glycol (PEG) (commercially branded Restoralax) in DMEM high glucose or Hanks’ Balanced Salt Solution (HBSS, Thermo Fisher). After two days of culture in antibiotic-free media, chips were treated with DMEM high glucose (∼350 mOsm/kg) or bowel prep solution made with DMEM high glucose (∼630 mOsm/kg) for 30 min at a 600 μL/h flow rate. Chips were disconnected from flow, and channel inlets and outlets were plugged with 200 μL filter tips as 50 μL of 10^6^ CFU/mL *Salmonella* Typhimurium was seeded into the apical channel in HBSS or bowel prep solution made in HBSS, depending on the condition. Bacteria infected statically for 1 h before filter tips were removed and chips were connected back to flow (120 μL/h) and mechanical deformation (10%, 0.15 Hz) in the incubator, with antibiotic-free DMEM high glucose with supplements. Media from all channels was sampled and spot-plated on LB-streptomycin agar plates at 4 h and 24 h post infection. After 24 h, chips were disconnected then all channels were washed thrice with PBS (Gibco), fixed in 4% paraformaldehyde (Electron Microscopy Sciences, 15710-S) diluted with DPBS (Gibco).

#### Paracellular permeability analysis

To assess paracellular permeability, 50 μg/mL of 3–5 kDa fluorescein isothiocyanate-dextran (FITC-dextran, Sigma-Aldrich) was added to the apical channel media on the day before *Salmonella* Typhimurium infection. Chips were run under a 600 μL/h flow rate for 5 min to flush out existing media, then run at a 100 μL/h flow rate for 3 h before samples from all channels were collected. Media was sampled prior to *Salmonella* Typhimurium infection to establish levels at baseline, after 4 h of infection, and after 24 h. The fluorescence intensities (490 nm/520 nm) of the top and bottom channel effluents were measured using a Synergy H1 plate reader (Biotek) and FITC-dextran concentration was estimated by plotting a log-log standard curve. The apical to basolateral flux of FITC-dextran was calculated according to the following equation using the Emulate Apparent Permeability Calculator EC004v1.0^123^:

$$P_{app}=-\frac{Q_{R}\;*\;Q_{D}}{SA\;*\;\left(Q_{R}+Q_{D}\right)\;}\;*\;\ln\;\left[1-\frac{C_{R,0}\;*\;\left(Q_{R}+Q_{D}\right)}{\left(Q_{R}\;*\;C_{R,0}+Q_{D}\;*\;C_{D,0}\right)}\right]$$

where *P*_*app*_ is the apparent permeability (cm/s); *SA* is the surface area of the sections of the channels that overlap (0.17 cm^2^); Q_R_ and Q_D_ are the fluid flow rates in dosing and receiving channels, respectively (cm^3^/s); and C_R,0_ & C_D,0_ are the recovered concentrations in the dosing and receiving channels, respectively.

#### Quantification of bacterial colonization in extraintestinal tissues

Liver, mesenteric lymph nodes, and spleen were collected in pre-weighed sterile tubes filled with 150 μL of PBS and weighed for final tissue mass. Tissues were homogenized at 300 Hz for 6 min using the TissueLyser II (QIAGEN). Homogenized samples were serially diluted at 1:10 in PBS, and all dilutions were spot-plated on both LB-streptomycin agar plates and Columbia Blood agar plates. Plates were incubated aerobically (in the case of *Salmonella* Typhimurium infection) or anaerobically (in the case of hIBD pathobiont translocation) overnight at 37°C. Single colonies were counted and back calculated to determine the absolute abundance of bacterial translocation to extraintestinal organs (liver, mesenteric lymph nodes, and spleen). Excess homogenized tissue was frozen at −80°C for downstream 16S rRNA sequencing.

#### Pathobiont growth measurements

1 μL of glycerol stocks of each of the 130 pathobionts isolated from UC patients were streaked on LB agar plates. A single colony was inoculated into 5 mL of liquid LB-streptomycin broth and grown for 17 h overnight aerobically and shaking at 200 rpm. All strains were subcultured 1:15 into LB broth in 2 mL 96 well plates, using a pipetting robot (INTEGRA VIAFLO384) and grown for 2 h at 37°C.^19^ Strains were then inoculated at a ratio of 1:75 into LB media with pH levels of 4.0, 5.5, 6.9, and 8.0, and osmolality levels of 400, 800, 1200, and 1800 mOsm/Kg, adjusted with PEG. The experiments were conducted in technical triplicates using a 384-well plate.

Pathobiont bacterial growth curves were obtained using a Biotek Synergy H1 plate reader at 37°C for 24 h either inside an anaerobic chamber (Coy Laboratories) with an atmosphere of 5% CO_2_, 5% H_2_, and 90% N2 (Linde Canada) or in aerobic conditions in parallel. The plates were shaken orbitally every 15 min following the collection of OD600 measurements. Background absorbance subtraction was performed from non-inoculated wells. The growth rates of the strains were determined using a previously published MATLAB package.^19^

#### *In vitro* hIBD microbiota growth

2 g of human stool inocula (hIBD1) were grown from the frozen fecal samples in 5 mL of MEGA medium^19^^,^^124^ aerobically overnight and subcultured 1:200 in fresh medium at baseline or adjusted to 800 mOsm/Kg with PEG. 16S rRNA sequencing was performed as described above (DNA Extraction, Library Preparation, and 16S rRNA Sequencing).

#### Human microbiota-associated mice

C57BL/6J germ-free mice were inoculated with 200 μL of prepped fecal samples from human donors at 6 weeks of age by oral gavage (ulcerative colitis, hIBD or healthy control, hHealthy, respectively) and maintained in isocages (Tecniplast) for 6 weeks to allow the microbiome to settle. Mice were used directly in experiments (Figures 6A–6H and S8C–S8H) or bred for offspring that were used (hIBD1, Figures S8A and S8B). Stool was sampled approximately daily.

#### Colitis disease activity index

Disease Activity Index (DAI) was measured daily based on the body weight loss, stool consistency and blood in stools using a previously validated approach.^39^^,^^40^^,^^41^ A higher DAI indicates worse disease state and inflammation. Body weight loss was scored as follows: score 0 for no body weight loss, score 1 for 0–5% weight loss, score 2 for 5–10% weight loss, and score 3 for >10% weight loss. Stool consistency was scored as follows: score 0 for normal fecal pellet, score 1 for soft but adherent in pellet shape, score 2 for loose stool, and score 3 for diarrhea. Fecal bleeding was scored as follows: score 0 for hemoccult negative, score 1 for hemoccult positive, score 2 for hemoccult positive with visual pellet bleeding, and score 3 for gross visual pellet and rectal bleeding. Behavior was scored as follows: score 1 for piloerect and some lethargy, score 2 for piloerect and lethargic with little interest in environment, score 3 for high lethargy with no interest in or response to environment or cage mates.

#### Mouse IBD DSS flare-up model

Humanized IBD mice received 2% Dextran Sodium Sulfate (DSS, ThermoFisher) in drinking water for 5 days to induce an acute colitis flare-up. Two days after DSS ended mice received bowel prep, or no treatment as a negative control. No vehicle control was used, as DSS treatment itself induces dehydration. This approach better reflects the clinical context of IBD patients recovering from colitis with or without bowel prep and avoids artificially altering hydration dynamics. Mice were sacrificed 24 h after bowel prep, and tissues and cecal contents were collected. Feces were collected at day 0 (start of DSS), day 8 (bowel prep) and day 9 (day of euthanasia). DAI was assessed daily as described above (Colitis disease activity index section). Tissue was homogenized and plated as described above (Quantification of Bacterial Colonization in Extraintestinal Tissues).

#### Colitis pathology scoring

H&E-stained sections of the cecal tip and distal colon (Hematoxylin & eosin tissue histology section) were assessed for pathology following previously published protocols for DSS-induced colitis.^59^ The following features of colitis were each scored on a scale of 0–3: immune cell infiltration extent and severity, goblet cell loss, crypt density, crypt hyperplasia, crypt abscesses, muscle thickening, and ulceration. The scores were summed, with the maximum pathological score being 24 per image. Two images of 1000 μm continuous mucosa were taken for each mouse, and were scored independently by two blinded scorers. Scores were then averaged for each mouse. Representative images shown in-text were selected for scoring close to the average score for that treatment group.

### Quantification and statistical analysis

#### Statistics in figures

Unless noted otherwise, all *in vivo* data points presented are one biological replicate, averaged across three technical replicates. For plating experiments where no CFUs were detected, the values were plotted at ½ the limit of detection (LoD) in log scale.

Statistical tests used are noted in figure captions. The Shapiro–Wilk test was first applied to test whether data were parametric. Comparisons between two treatment groups were performed with the Student’s *t* test for parametric data, or a Wilcoxon Rank-Sum Test for non-parametric data. For comparisons between multiple groups, parametric data were analyzed using a one-way ANOVA with Tukey’s post hoc test, and non-parametric data were analyzed Kruskal-Wallis followed by Dunn’s post hoc test. Comparisons within a group at different timepoints were analyzed with a Friedman test followed by Nemenyi post hoc test. No outlier detection was performed prior to statistical analysis and plotting. *p* > 0.05; ns (not significant, not shown), *p* < 0.05; ∗, *p* < 0.01; ∗∗, *p* < 0.001; ∗∗∗, *p* < 0.0001; ∗∗∗∗.

## Supplemental information

Document S1. Figures S1–S8

Table S1. Mouse cecal RNA-seq data and statistics, collected at baseline and 6 h post-bowel prep, refers to Figures S1G–S1I (A) “rna-seq-raw_counts” summarizes the raw gene expression counts from the extracted RNA under baseline conditions and 6 h post-bowel prep. (B) “DESeq_lfc_Z″ summarizes the results table generated from the DESeq2 analyses and the Z-scores across multiple conditions and time points. **Sheet “rna-seq-raw_counts”:** this table summarizes the raw gene expression counts from the extracted RNA.

• CC_1_S59ReadsPerGene.out.tab-CC_6_S64ReadsPerGene.out.tab: raw gene expression counts under baseline conditions for mice 1–6.
• CC_7_S65ReadsPerGene.out.tab-CC_12_S70ReadsPerGene.out.tab: raw gene expression counts at 6 h post-treatment for mice 1–6.
• Gene: the official symbol for the gene.
• Description: a brief description of the gene’s function or associated protein.
• Baseline_x: the average normalized counts, normalized by sequencing depth across all samples for mice 1–6 under baseline conditions.
• 6h_x: the average normalized counts, normalized by sequencing depth across all samples for mice 1–6 at 6 h post treatment.
• Log2FoldChange_x: indicates how much the gene expression changed between the samples collected at 6 h post-treatment and the samples collected at baseline. This value is reported on a log2 scale.
• lfcSE_x: the standard error estimate for the log2 fold change estimate for the samples collected at 6 h post-treatment.
• pvalue_x: *p* value of the test for the gene for the samples collected at 6 h post-treatment.
• padj_x: Adjusted *p* value for multiple testing for the gene for the samples collected at 6 h post-treatment.
• mean_Baseline, mean_6h: mean normalized counts for each respective condition, averaged across all replicates.
• Z_Baseline_x: Z-scores representing gene expression levels under baseline conditions for mice 1–6.
• Z_6h_x: Z-scores for gene expression at 6 h post-treatment for mice 1–6.
• Z_Baseline_mean, Z_6h_mean: mean Z-scores for each respective condition, averaged across all replicates.

Table S2. Papp calculations, from all channels at baseline, 4 h, and 24 h post-infection, refers to Figure S6H **(A) Sheet “Papp_Calculator”** contains the template of the Emulate Papp calculator^108^ used in other sheets. (**B) Sheet “baseline”:** this sheet shows the Papp calculations for samples collected at baseline. (**C) Sheet “4hpi”:** this sheet shows the Papp calculations for samples collected at 4 h post-infection. (**D) Sheet “24hpi”:** this sheet shows the Papp calculations for samples collected at 24 h post-infection.

Table S3. Outputs from GSEA analysis, refers to Figures 5C and 5D

• ID: Gene Ontology (GO) identifier, a unique accession for a biological process.
• Description: plain-text name of the GO term associated with the ID.
• setSize: number of genes from the dataset that fall within the given GO term gene set.
• enrichmentScore: raw enrichment score (ES) reflecting the degree to which genes in a GO term are overrepresented at the top or bottom of the ranked gene list. Positive ES means enrichment in upregulated genes; negative ES means enrichment in downregulated genes.
• NES: normalized enrichment score (NES) is the enrichment score normalized for gene set size, allowing comparisons across gene sets of different sizes.
• pvalue: nominal *p* value assessing significance of the observed enrichment score by permutation testing.
• p.adjust: multiple hypothesis testing–adjusted *p* value (via Benjamini–Hochberg correction).
• qvalue: estimated false discovery rate (FDR) for the enrichment result.
• rank: position in the ranked gene list where the enrichment score was maximal (peak enrichment).
• leading_edge: subset of genes that contribute most strongly to the enrichment signal. Reported as percentages of “tags” (genes in the set contributing), “list” (portion of ranked list contributing), and “signal” (relative strength of enrichment).
• core_enrichment: specific genes from the dataset that belong to the leading edge subset, reported as Entrez Gene IDs.

Document S2. Article plus supplemental information
