# Supplementary material for: In mouse and in vitro models, bowel preparation promotes pathogen colonization, translocation, and exacerbation of inflammation
Source: Cell Rep Med. 2025 Dec 22;7(1):102517. doi: 10.1016/j.xcrm.2025.102517 (PMC12866094; doi:10.1016/j.xcrm.2025.102517)
Supplement: Document S1. Figures S1–S8 [file mmc1.pdf]

Cell Reports Medicine, Volume *7*

Supplemental information

In mouse and *in vitro* models, bowel preparation promotes pathogen colonization, translocation, and exacerbation of inflammation

Charlotte A. Clayton, Imogen Porter, Brian D. Deng, Giselle McCallum, Apsara Srinivas, Claire Sie, Jerry Y. He, Alexander D. Pei, Dominique Tertigas, Deanna M. Pepin, Touran Fardeen, Katharine M. Ng, Sidhartha R. Sinha, Michael G. Surette, Bruce A. Vallance, and Carolina Tropini

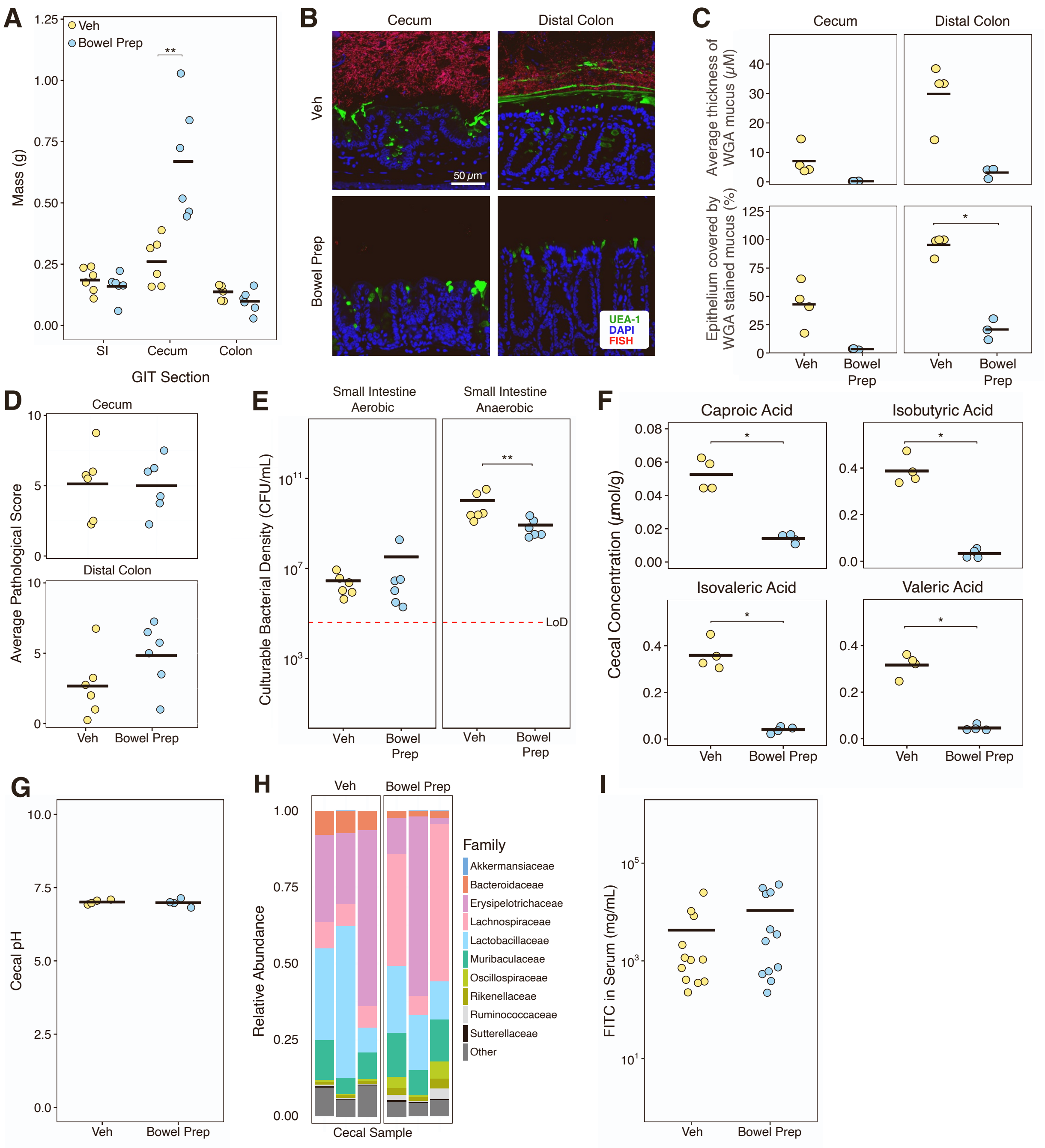

Figure S1. Bowel prep does not lead to overt pathology in the mouse gut at 6 hours post procedure, despite changes in cecal mass, mucus layer thickness, and short-chain fatty acid (SCFA) levels. Related to Figure 1.

(A) The mass of the cecum, the small intestine and colon measured in bowel prep- vs vehicle-treated mice (Veh *n*=6, Bowel Prep *n*=6). (B) Representative confocal micrographs of the cecal tip and distal colon (stained by FISH [*red*]) as well as UEA-1 stained mucus [*green*] following bowel prep. (C) Average thickness of mucus (*top*) and percentage of the epithelium covered (*bottom*), quantified using WGA fluorescence in bowel prep- vs vehicle-treated mice (Veh *n*=4, Bowel Prep *n*=3). (D) Average pathological scores of the cecum and distal colon in bowel prep- and vehicle-treated mice (Veh *n*=6, Bowel Prep *n*=6). (E) Bacterial loads in the small intestine (SI), as measured by culture under aerobic and anaerobic conditions (Veh *n*=6, Bowel Prep *n*=6). (F) Cecal SCFAs abundance in bowel prep- and vehicle-treated mice (Veh *n*=4, Bowel Prep *n*=4). (G) Cecal pH in bowel prep- and vehicle-treated mice (Veh *n*=4, Bowel Prep *n*=4). (H) 6 hours post-treatment, 16S rRNA sequencing of the contents of the cecum (Veh *n*=3, Bowel Prep *n*=3). These data are independent of those in Fig. 1G. (I) Gut permeability measured by FITC-Dextran movement from the gut into serum (Veh *n*=12, Bowel Prep *n*=12, two independent replicates). **Statistics**: Differences between treatment groups were analyzed using a Wilcoxon ranked-sum test. p > 0.05; ns (not significant, not shown), p < 0.05; *, p < 0.01; **, p < 0.001; ***, p < 0.0001; ****.

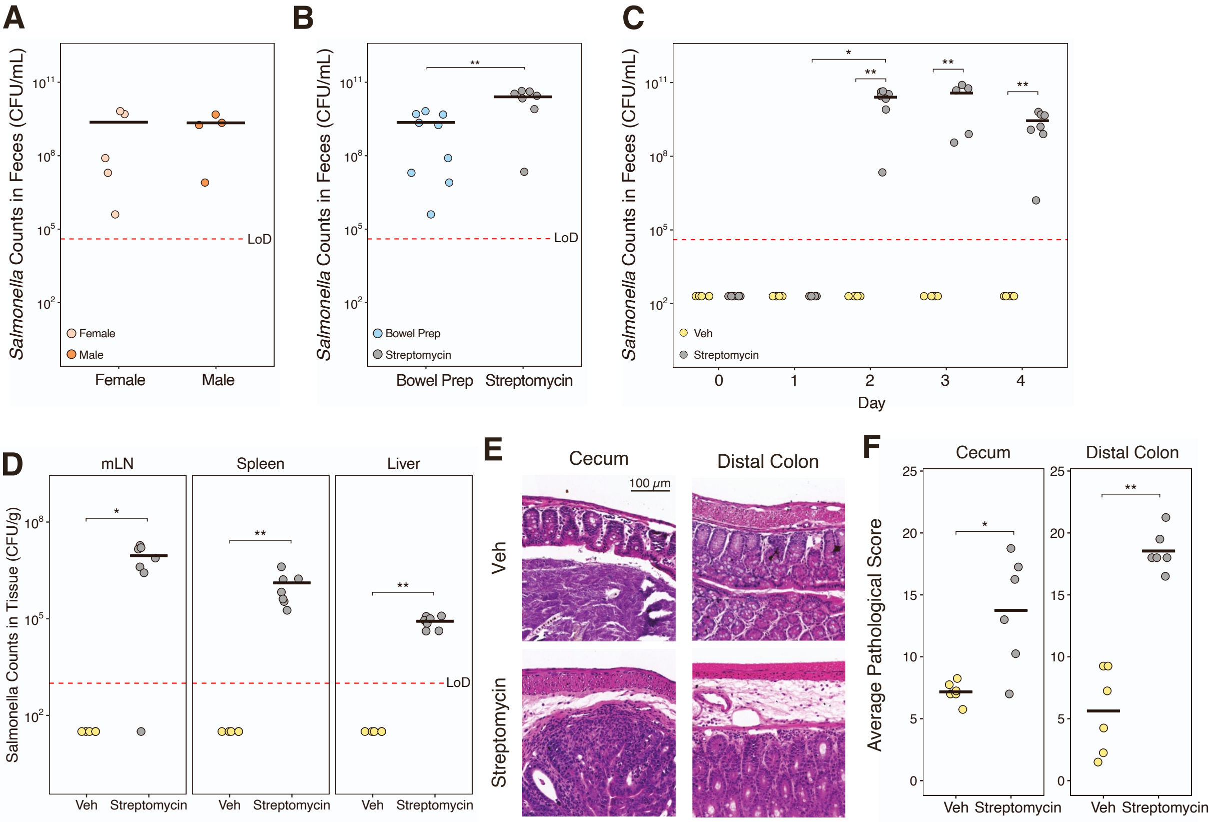

Figure S2. Comparison of two mouse models of *Salmonella* Typhimurium—pre-treatment with bowel prep or streptomycin—shows high levels of *Salmonella* Typhimurium burdens and robust translocation. Related to Figure 2.

(A) Fecal *Salmonella* Typhimurium levels 3-days post inoculation (Female *n*=5, Male *n*=4). Differences between treatment groups were analyzed using a Wilcoxon ranked-sum test. (B) Fecal *Salmonella* Typhimurium levels inoculated 6 hours post bowel prep (from **Fig. 2C**) and in streptomycin-treated mice 24-hours post inoculation (Bowel Prep *n*=9, Streptomycin *n*=7). Differences between treatment groups were analyzed using a Wilcoxon ranked-sum test. (C) Fecal *Salmonella* Typhimurium levels in vehicle- vs streptomycin-treated mice up to four days after inoculation (Veh *n*=5, Streptomycin *n*=7). (D) *Salmonella* Typhimurium translocation from the gut to the mLN, liver, and spleen 72 hours after inoculation (Veh *n*=5, Streptomycin *n*=7). (E) H&E-stained sections of the cecum and distal colon in streptomycin- and vehicle-treated mice three days after inoculation with *Salmonella* Typhimurium. (F) Histopathological scoring of the distal colon and cecum in streptomycin- and vehicle-treated mice in cecum and distal colon at three days after inoculation with *Salmonella* Typhimurium (Veh *n*=6, Streptomycin *n*=6). **Statistics:** Comparisons between treatment groups were measured using the Wilcoxon ranked-sum test, and within groups at different timepoints with a Friedman test followed by Nemenyi post-hoc test. $p > 0.05$; ns (not significant, not shown), $p < 0.05$; *, $p < 0.01$; **, $p < 0.001$; ***, $p < 0.0001$; ****.

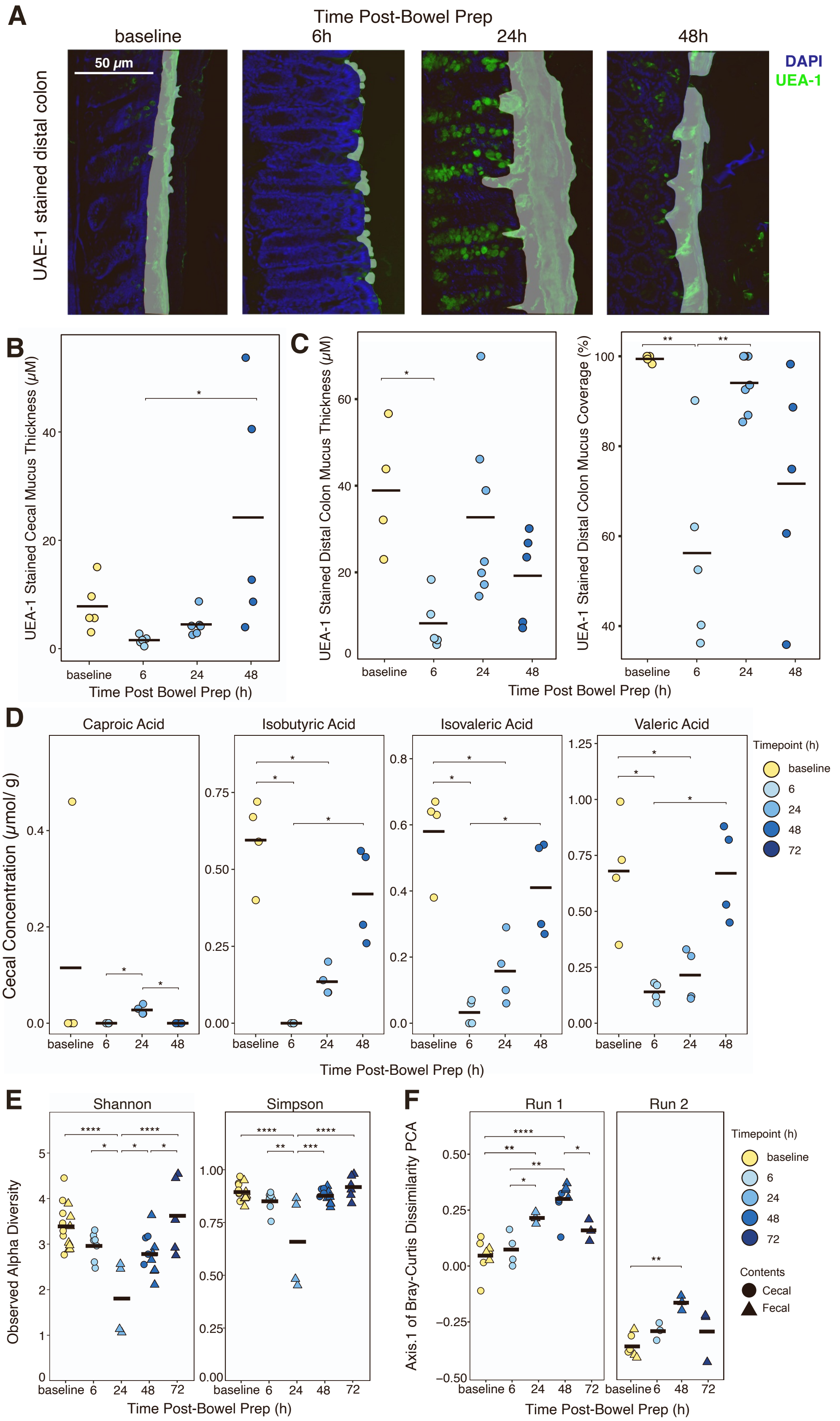

Figure S3. In the mouse cecum, short-chain fatty acid (SCFA) levels take longer to recover than the mucus layer and microbiota diversity after bowel prep. Related to Figures 1 and 3.
(A) Representative confocal images of the distal colon stained with DAPI which indicates host DNA (*blue*) and UEA-1 which indicates mucus (*green*). Contour lines generated in BacSpace software show the measured boundary used to calculate mucus thickness and coverage. (B) Mucus thickness in the cecum (baseline, 6 h, 48 h

n=5, 24 h *n* =6, 3 independent experiments). (C) Mucus thickness and coverage in the distal 6 hours after bowel prep. (D) SCFA levels at different timepoints after PEG treatment (each timepoint *n*=4). (E) Additional alpha diversity metrics in the cecal and fecal microbiome, determined from 16S rRNA sequencing (baseline *n*=14, 6h *n*=7, 24h *n*=4, 48h *n*=11, 72h *n*=6, 4 independent experiments). (F) Bray Curtis dissimilarity values for the cecal and fecal microbiome, determined from 16S rRNA sequencing. Two Illumina sequencing runs are shown, with separate biological samples (Run 1 baseline *n*=8, 6h *n*=4, 24h *n*=4, 48h *n*=8, 72h *n*=3; Run 2 baseline *n*=6, 6h *n*=3, 48h *n*=3, 72h *n*=3). **Statistics:** Differences between timepoints were analyzed using a one-way ANOVA with Tukey's post-hoc test for multiple comparisons (B,E,F) or with Kruskal-Wallis followed by Dunn's post-hoc test (D). $p > 0.05$; ns (not significant, not shown), $p < 0.05$; *, $p < 0.01$; **, $p < 0.001$; ***, $p < 0.0001$; ****.

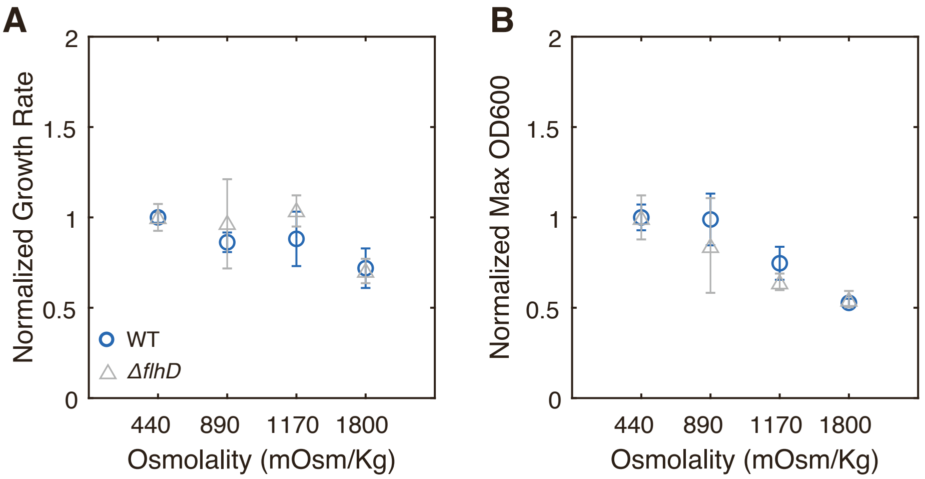

Supplemental Figure 4. In culture, growth of the *Salmonella* Typhimurium non-motile *ΔflhD* mutant is robust under high osmolality conditions. Related to Figure 4.
(A) Normalized maximum growth rate and (B) normalized maximum optical density (OD600) of wild type and *ΔflhD Salmonella* Typhimurium in aerobic conditions and media of varying osmolalities, adjusted with PEG (each osmolality & strain *n*=4). **Statistics:** Differences between timepoints and strains were analyzed using a two-way ANOVA with Tukey's post-hoc test for multiple comparisons.

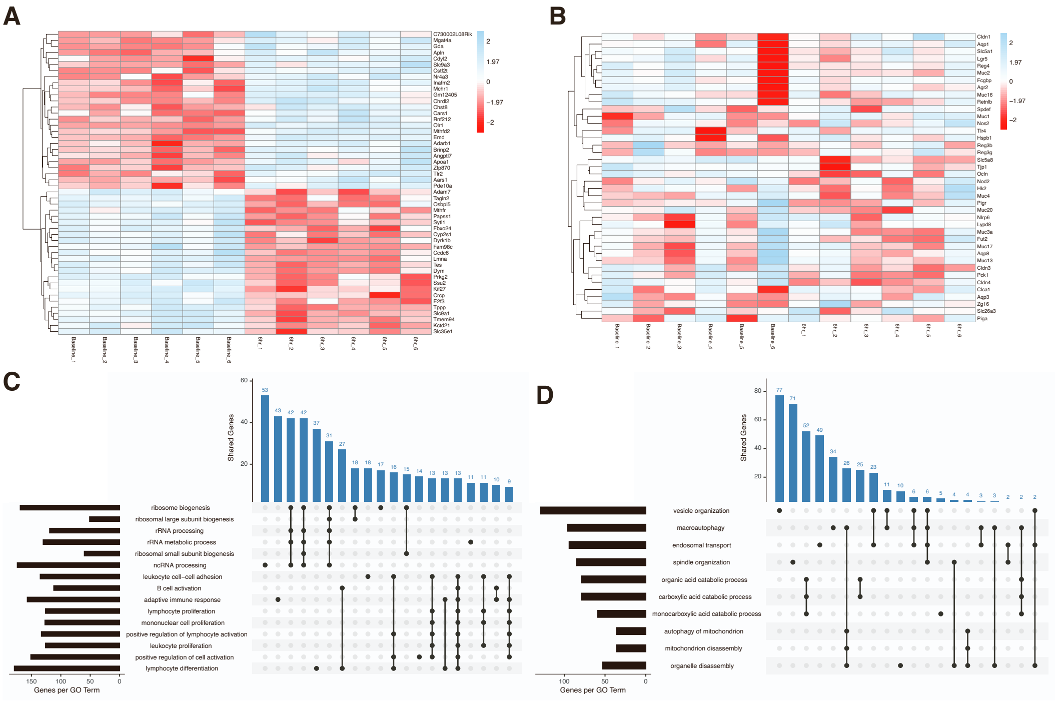

Supplemental Figure 5. Bowel prep in mice does not lead to clear clustering of individual gene expression profiles, but enriched Gene Ontology (GO) terms share core enrichment genes. Related to Figure 5. (A) Heatmap of the top 50 genes ranked by absolute Z score across all samples from cecal tip tissue at baseline and 6 hours after bowel prep. (B) Heatmap of 40 selected genes of interest, relating to mucus production, immune system activation, and tight junction formation. (C–D) Upset plots showing the overlap of leading-edge subset genes among significantly enriched Gene Ontology (GO) Biological Process terms identified by GSEA at 6 hours post–bowel prep vs baseline (Table S3). The bar plot on the left indicates the number of core enrichment genes of each GO term; the bar plot above indicates the number of shared core enrichment genes between the combinations of GO terms connected in the matrix below. Only the top 20 shared gene overlaps are shown. (C) Significantly positively enriched GO terms (NES > 0) show shared gene membership among pathways related to protein synthesis and immune activation. (D) Significantly negatively enriched GO terms (NES < 0) show overlapping genes among pathways involved in organelle degradation and catabolic processes. **Statistics:** p > 0.05; ns (not significant, not shown), p < 0.05; *, p < 0.01; **, p < 0.001; ***, p < 0.0001; ****.

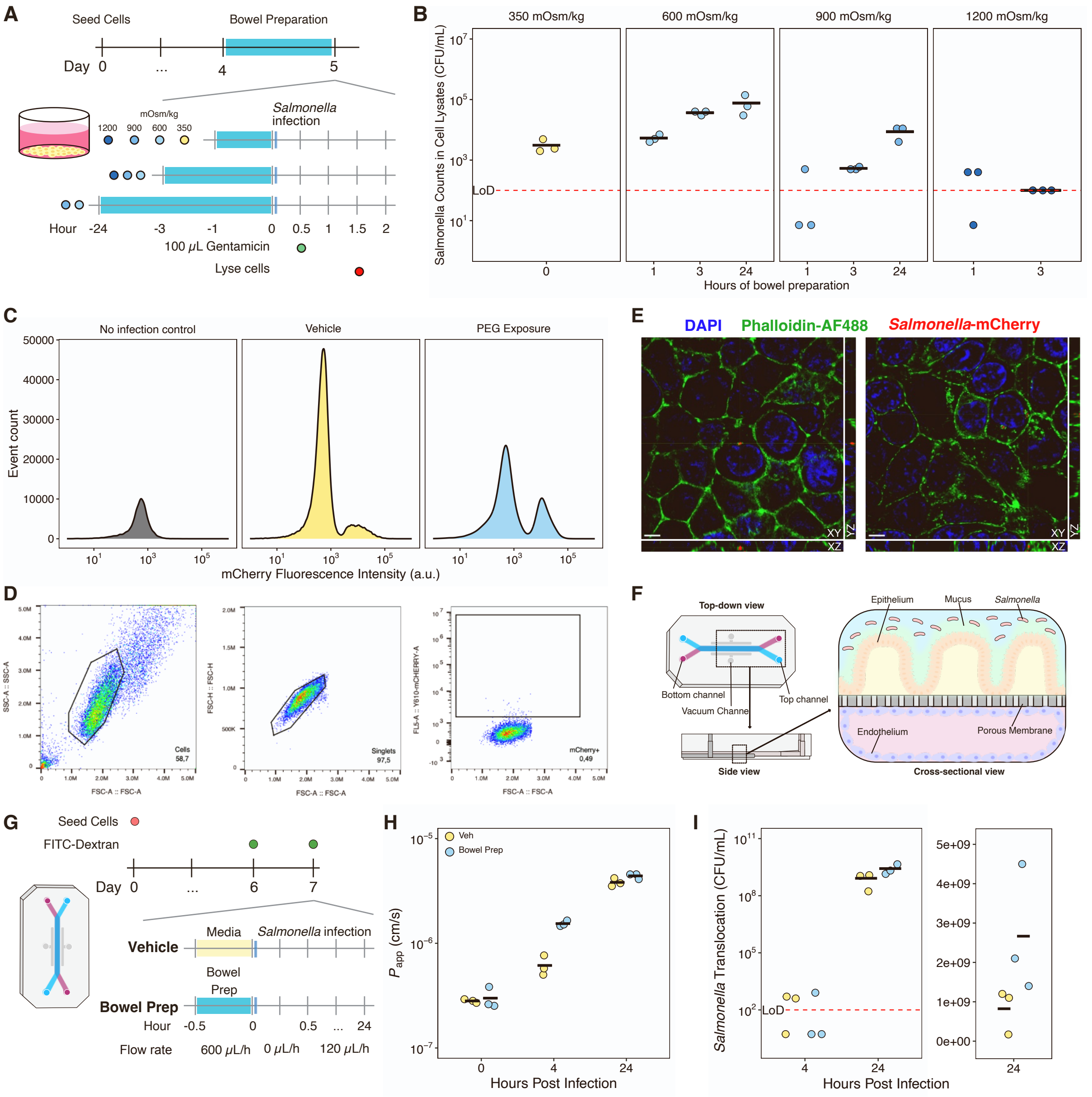

Supplemental Figure 6. PEG exposure promotes *Salmonella* Typhimurium invasion in intestinal epithelial monolayers and increased permeability in a gut-on-a-chip model. Related to Figure 5.

(A) Schematic of bowel prep dosage experiment in HT-29 monolayers. Cells were exposed to media adjusted to 600, 900, and 1200 mOsm/kg using PEG for 1, 3, or 24 hours, then infected with *Salmonella* Typhimurium. After 30 minutes of infection, cells were treated with 100 µg/mL gentamicin for 1 hour, then lysed for spot plating. (B) HT-29 monolayers were treated at 600 or 900 mOsm/kg and infected with *Salmonella* Typhimurium; invasion was quantified by spot plating serial dilutions of cell lysates (*n*=3 per condition per timepoint). Each dot represents one monolayer, with mean CFU/mL shown as black bars. (C) Representative flow cytometry histograms showing the mCherry fluorescence intensity event counts of the uninfected control (left, grey), infected vehicle–treated cells (middle, yellow), and infected bowel prep–treated cells (right, blue), from the experiment done in Fig. 5E. (D) Flow cytometry gating strategy to identify cells infected with mCherry-expressing *Salmonella* Typhimurium. The threshold for mCherry-positive signal was set to 1%> of events of the uninfected control population, as shown. (E) Orthogonal projections of HT-29 cells from Fig. 5J confocal micrographs stained with DAPI (blue) to visualize nuclei and Alexa Fluor 488–conjugated phalloidin (green) to label filamentous actin show that mCherry-*Salmonella* Typhimurium (red) is localized intracellularly. The XZ orthogonal view is shown at the bottom, and the YZ orthogonal view is shown on the right. The scale bars are 5 µm. (F) Schematic of gut-on-a-chip experiment designed to simulate the human gut environment, with top down, side, and cross-sectional views. (G) Schematic of gut-on-a-chip bowel prep infection model. Chips were exposed to vehicle (350 mOsm/kg) or bowel prep (600 mOsm/kg) treatment at high flow rate (600 µL/h), infected statically with *Salmonella* Typhimurium for 30 minutes, then exposed to fluid flow (120 µL/h) until 24 hours post infection. (H) Apparent permeability (Papp) of the gut epithelial layer on the gut-on-a-chip,

as measured by FITC-dextran permeability assay 4 hours after exposure to *Salmonella* Typhimurium (Veh *n*=3, Bowel Prep *n*=3). Each dot corresponds to one chip, with mean Papp for a treatment group/time period shown as black bars. (I) Mean *Salmonella* Typhimurium translocation from the luminal side (top channel) to pass the endothelial layer (bottom channel) 24 hours post-pathogen exposure (Veh *n*=3, Bowel Prep *n*=3). Both the log scale (left) and the linear scale (right) are shown. Each dot corresponds to one chip, with mean CFU/mL shown as black bars. **Statistics:** For all comparisons, statistical significance was assessed using the Wilcoxon ranked-sum test. p > 0.05; ns (not significant, not shown), p < 0.05; *, p < 0.01; **, p < 0.001; ***, p < 0.0001; ****.

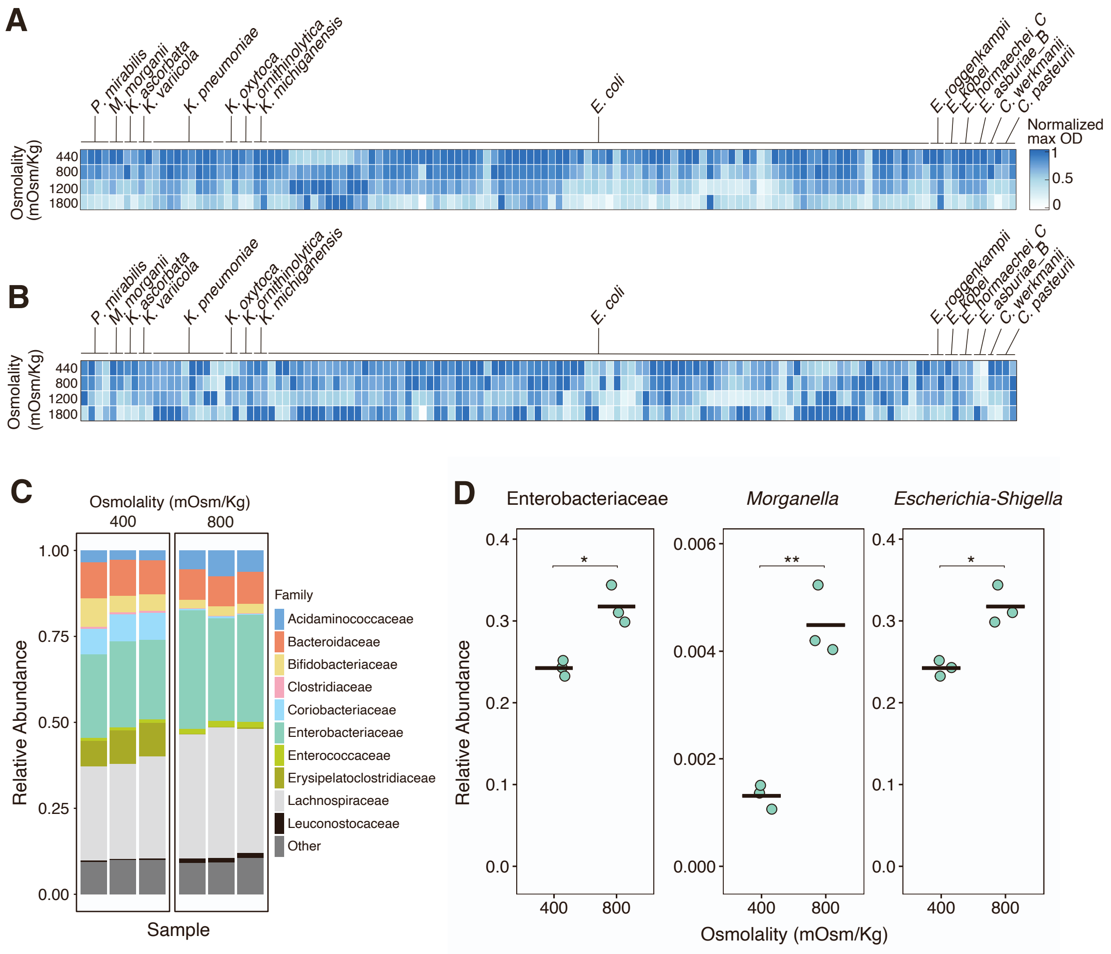

Supplemental Figure 7. Potential pathobionts from patients with inflammatory bowel disease are resilient to osmotic perturbation *in vitro* and can translocate and persist in the mesenteric lymph nodes of germ-free mice after bowel prep. Related to Figure 6.

(A) Growth of 130 potential pathobionts isolated from patients with ulcerative colitis and cultured under anaerobic conditions at osmotic stress levels meeting or exceeding those found in the mouse gut after bowel prep (~750 mOsm/Kg). Normalized carrying capacity was measured at maximum OD600 after bacteria were cultured under anaerobic conditions for 24 hours, and cultures were grown at 400, 800, 1200, and 1800 mOsm/Kg, adjusted with NaCl. (B) Potential pathobionts from (A) cultured in aerobic conditions. (C) 16S rRNA sequence data from a fecal sample obtained from an individual with ulcerative colitis cultured under osmotic stress levels simulating normal (400 mOsm/Kg) and (PEG)-treated (800 mOsm/Kg) conditions. (D) Relative abundance of the Enterobacteriaceae family and the genera *Morganella* and *Escherichia-Shigella* from (B) (each osmolality *n*=3). **Statistics:** Differences between treatment groups were analyzed with t-tests. p > 0.05; ns (not significant, not shown), p < 0.05; *, p < 0.01; **, p < 0.001; ***, p < 0.0001; ****.

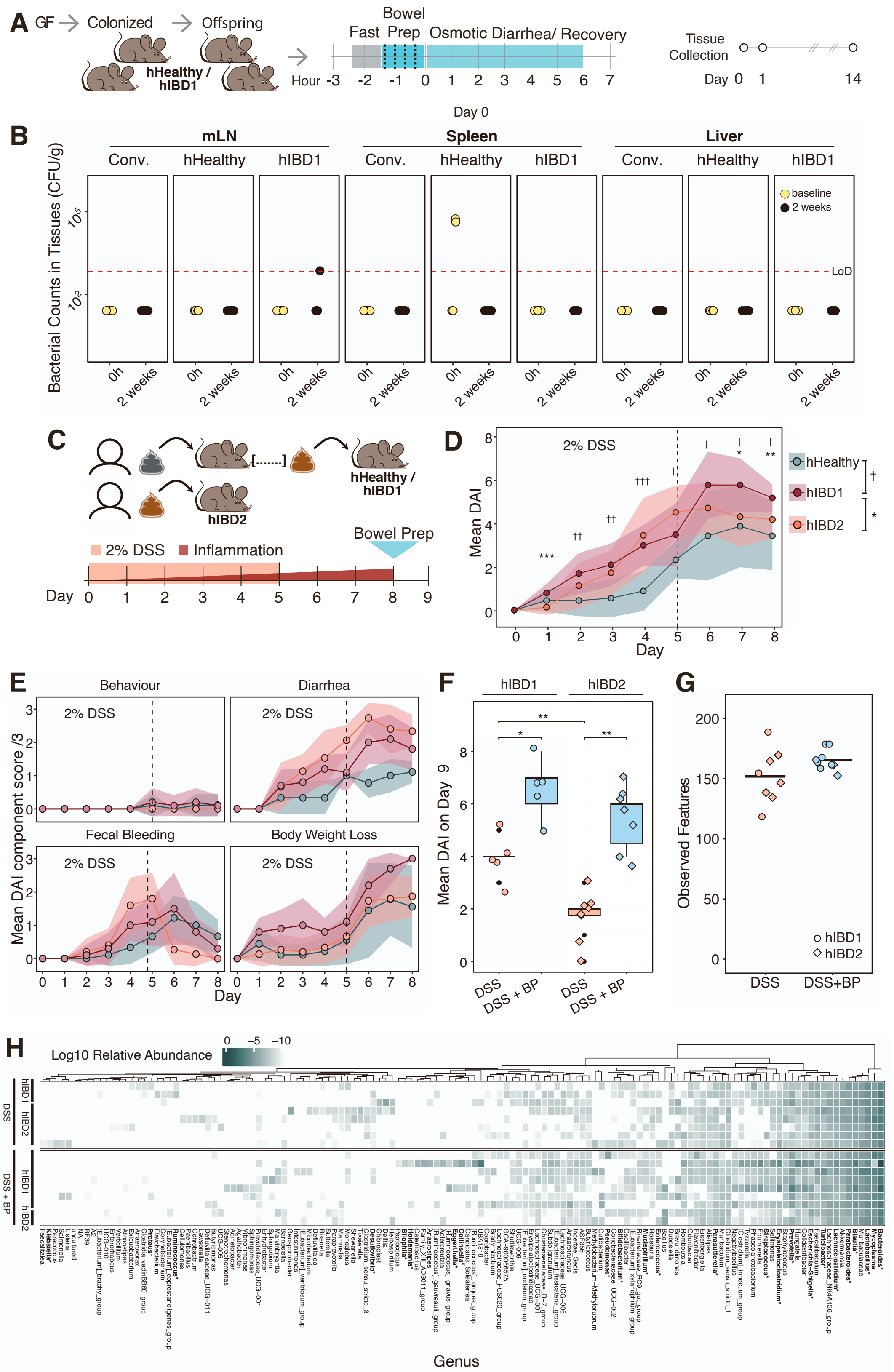

Supplemental Figure 8. Germ-free mice humanized with microbiota from patients with inflammatory bowel disease experience greater chemically-induced colitis disease than those humanized with a healthy microbiota, in a microbiota-dependent manner. Related to Figure 6.

(A) Schematic of germ-free (GF) C57BL/6J mice colonized with a fecal sample from a patient with ulcerative colitis (hIBD1) or without the condition (hHealthy) and then bred for experimental use. Mouse offspring 8-12 weeks of age were bowel prepped and sampled 2 weeks post-bowel prep. (B) Anaerobic bacterial translocation

measured in extraintestinal organs, mesenteric lymph nodes (mLN), spleen, and liver, at baseline and 2 weeks post-bowel prep in conventional (Conv.) mice, hHealthy mice, and hIBD mice after bowel prep (Conv. each tissue: baseline n=5, 2 weeks n=7. hHealthy each tissue: baseline n=4, 2 weeks n=5. hIBD1 mLN: baseline n=4, 2 weeks n=4. hIBD Spleen & Liver: baseline n=5, 2 weeks n=4.). (C) Schematic depicting humanisation of GF CBL57/6J mice with fecal samples from mice previously humanized using healthy (hHealthy) or ulcerative colitis (hIBD1) patient samples, or with fecal samples directly from a patient with ulcerative colitis (hIBD2). Mice were treated with 2% dextran sodium sulphate (DSS) in drinking water for 5 days to stimulate a colitis flare-up. (D) Mean disease activity index (DAI) was assessed daily within the three microbiota. Statistical comparisons between hIBD1 & hIBD2, and hIBD1 & hHealthy, are denoted by * and † respectively. (E) Detailed breakdown of the DAI components show that colitis disease symptoms are dependent on mouse microbiota. Statistics not shown. (F) On day 9, 24 hours after bowel prep, both hIBD microbiota groups show an increase in DAI compared to mice of the same microbiota that did not receive the treatment. (G) Stool 16S rRNA sequencing on day 9. (H) 16S rRNA sequencing of bacteria translocated to the mLN of mice from (C-G). Pathobionts associated with IBD[6,45–59] are bolded. **Statistics:** For all comparisons, statistical significance was assessed using the Wilcoxon ranked-sum test. $p > 0.05$; ns (not significant, not shown), $p < 0.05$; *, $p < 0.01$; **, $p < 0.001$; ***, $p < 0.0001$; ****.
